# Supplementary material for: Lipid nanoparticles enable mRNA delivery to diverse cell types of the inner Retina
Source: Mol Ther Nucleic Acids. 2026 Jun 30;37(3):102996. doi: 10.1016/j.omtn.2026.102996 (PMC13382181; doi:10.1016/j.omtn.2026.102996)
Supplement: Document S1. Figures S1–S7 [file mmc1.pdf]

OMTN, Volume *37*

Supplemental information

Lipid nanoparticles enable mRNA delivery

to diverse cell types of the inner Retina

Sumit Biswas, Eleonora Carpentiero, Nermina Xhaferri, Paula Streckenbach, Vijay Renigunta, and Moritz Lindner

Supplemental Information

Table S1: Summary of reported metrics in this study

Experimental Condition	Route of delivery	Metric	Degenerate explants	Healthy explants	Healthy explants (ILM-peeled)
Late transfection	Subretinal	$mCherry^+$ cells/FOVs	28.25 [±2.60] (n=3 biological replicates, 8 FOVs)	Not assessed	Not assessed
Acute transfection	Subretinal	$mCherry^+$ cells/FOVs	34.889 [±4.01] (n=3 biological replicates, 9 FOVs)	Not assessed	Not assessed
Late transfection	Subretinal	$GFAP^+$ $mCherry^+$ cells/FOVs	22.375 [±2.43] (n=3 biological replicates, 8 FOVs)	Not assessed	Not assessed
Acute transfection	Subretinal	$GFAP^+$ $mCherry^+$ cells/FOVs	26.33 [±3.76] (n=3 biological replicates, 9 FOVs)	Not assessed	Not assessed
Late transfection	Subretinal	$GFAP^-$ $mCherry^+$ cells/FOVs	5.875 [±0.64] (n=3 biological replicates, 8 FOVs)	Not assessed	Not assessed
Acute transfection	Subretinal	$GFAP^-$ $mCherry^+$ cells/FOVs	8.555 [±0.88] (n=3 biological replicates, 9 FOVs)	Not assessed	Not assessed
Acute transfection	Intravitreal	$mCherry^+$ cells/FOVs	13.444 [±1.79] (n=3 biological replicates, 27 FOVs)	4.583 [±0.80], (n=3 biological replicates, 24 FOVs)	8.515 [±0.92] (n=3 biological replicates, 33 FOVs)
Acute transfection	Subretinal	$mCherry^+$ cells/FOVs	32.444 [±4.16] (n=3 biological replicates, 27 FOVs)	7.90 [±0.82] (n=3 biological replicates, 28 FOVs)	Not assessed
Acute transfection	Intravitreal	GFAP MFI (A.U.)	15703.4 [±1019.91] (n=3 biological replicates, 27 FOVs)	1646.92 [±238.56] (n=3 biological replicates, 24 FOVs)	2870.45 [±302.29] (n=3 biological replicates, 34 FOVs)
Acute transfection	Subretinal	GFAP MFI (A.U.)	14850.1 [±1593.71] (n=3 biological replicates, 27 FOVs)	2653.59 [±267.44] (n=3 biological replicates, 29 FOVs)	Not assessed
Acute transfection	Untransfected controls (NTC)	GFAP MFI (A.U.)	8756.56 [±487.24] (n=3 biological replicates, 25 FOVs)	1304.04 [±174.57] (n=3 biological replicates, 24 FOVs)	Not assessed

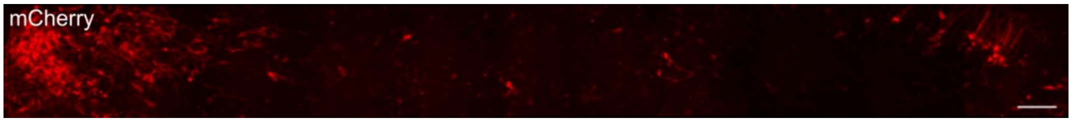

Figure S1: Comprehensive visualization of cmRNA^{mcherry}m^{1}Ψ$_{1.0}$m^{5}C$_{1.0}$-LNP transfected region in retina.

Retinal explants from rd1 retina-degenerate mice transfected by quasi-subretinal delivery (150 ng/μL). Multiple overlapping micrographs were stitched into a composite image to allow the assessment of transfection throughout the retinal explant. Scale bar: 150 μm.

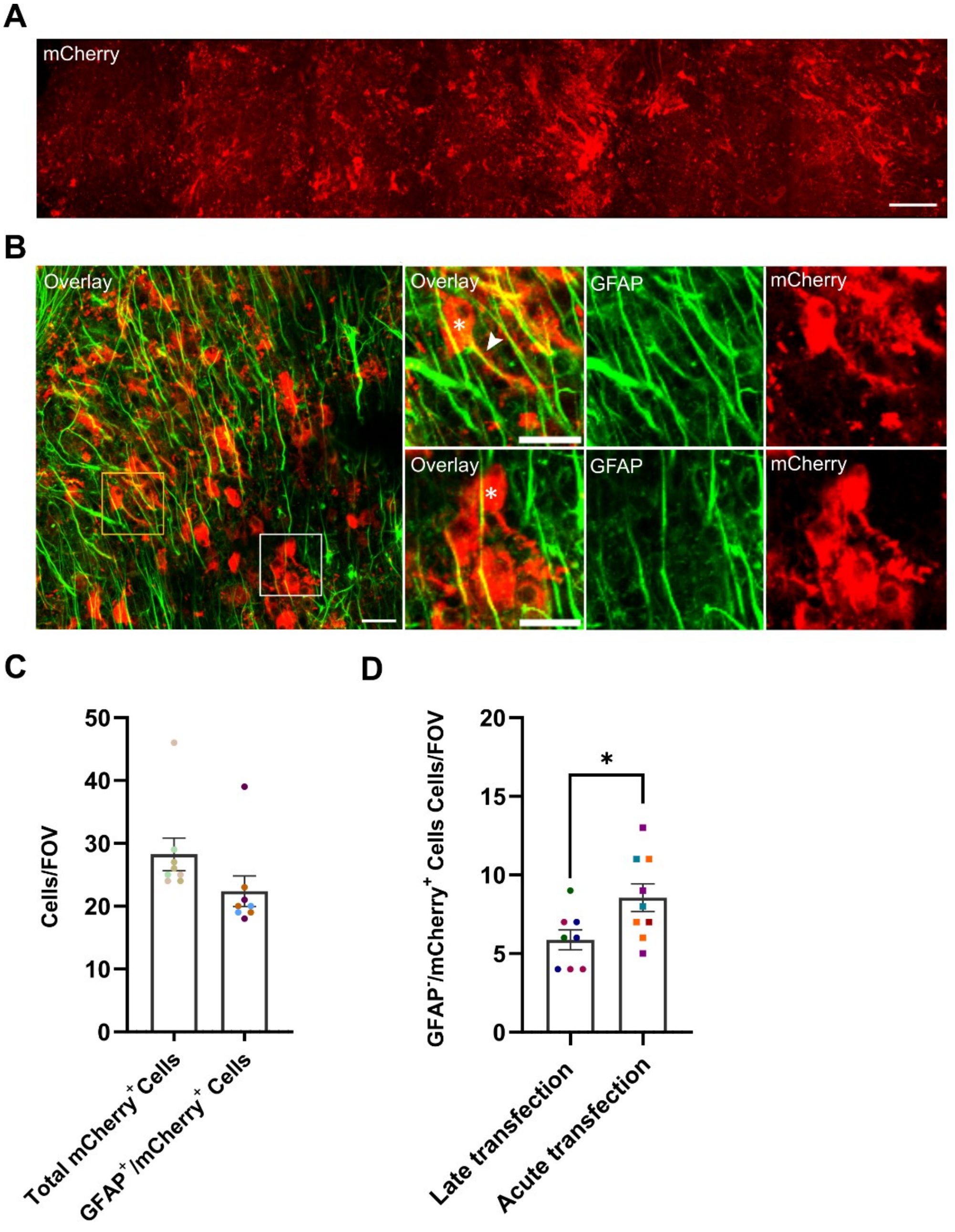

Figure S2: Late transfection in degenerate retinal explants using cmRNA^{mcherry}m$^{1}\Psi_{1.0}$m^{5}C$_{1.0}$-LNP

Degenerate retinal explants from rd1 mice transfected by quasi-subretinal delivery using LNPa two days after explantation. (A) Stitched composite image was generated from multiple overlapping micrographs to visualize the full extent of transfection across the explant. (B) Representative micrographs of explants immunostained for mCherry (red) and GFAP (green). Arrowhead represents the overlapping transfected cells and Muller glia process. Asterisk depicts a GFAP^{-}/mCherry^{+} cell (C) Number of total mCherry^{+} cells and subset of GFAP^{+}/mCherry^{+} cells per field of view (FoV) after late transfection, assessed 24 hours post-transfection. (D) Quantification of GFAP^{-}/mCherry^{+} cells per FoV, representing the complementary subset of the total mCherry^{+} cells after late and acute

transfection. Dots represent data acquired from an individual FoV, colours represent individual biological replicates (3 per condition). A FoV represents a single confocal micrograph (212.55 µm x 212.55 µm). Note that GFAP does not stain the somata of Müller glia but rather their processes, hence, cells were graded as $GFAP^+$ based on their processes. Scale bar: A= 150 µm, B= 20 µm, B upper and lower panel= 10 µm. Data obtained from 3 independent biological replicates per condition. Statistical significance of the data in panel 2D was performed using an unpaired t test. Unless specified otherwise, data are shown as mean ± SEM. *p*-values are reported as ns (non-significant), * $p < 0.05$, ** $p < 0.01$, *** $p < 0.001$ or **** $p < 0.0001$.

A

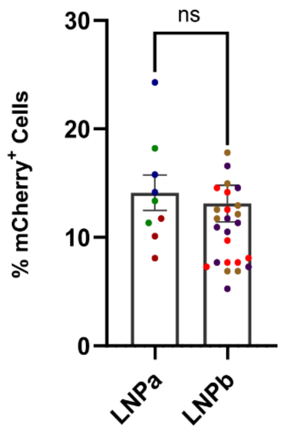

Figure S3: Transfection efficiency of LNPa and LNPb encapsulated cmRNA^{mcherry}m$^{1}\Psi_{1.0}$m^{5}C$_{1.0}$.

Degenerate retinas (rd1, C3H; A) were transfected with LNPa and LNPb using subretinal delivery under acute transfection conditions. Transfection efficiency was assessed 24 h post-transfection. Statistical significance was determined using Mann-Whitney U test. To enhance visualization, the y-axis in panel A is truncated; two values exceed the displayed range and are not shown but were included in the statistical analysis. Unless specified otherwise, data are shown as mean ± SEM. *p*-values are reported as ns (non-significant), * $p < 0.05$, ** $p < 0.01$, *** $p < 0.001$ or **** $p < 0.0001$.

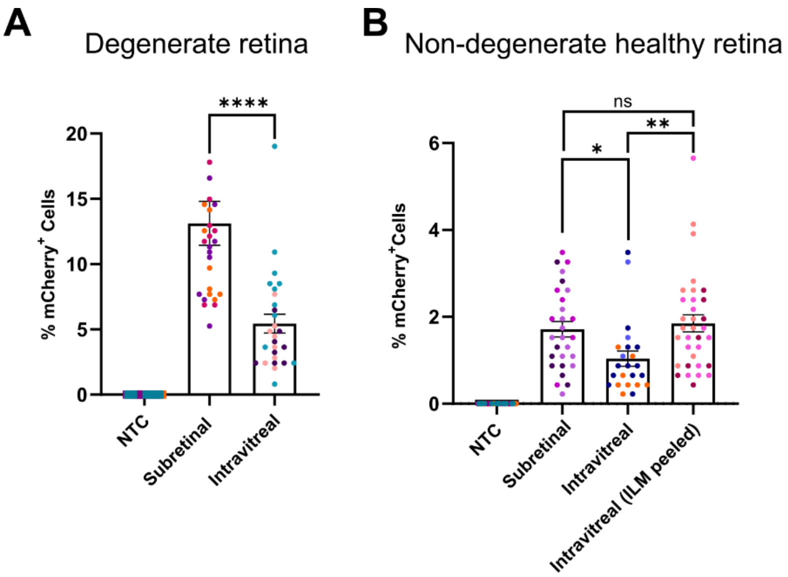

Figure S4: Transfection efficiency in degenerate and non-degenerate healthy retinal explants.

Quantification of transfection efficiency of cmRNA^{mcherry}m$^1\Psi_{1.0}$m^{5}C$_{1.0}$-LNP in retinal explants from degenerate (rd1, C3H; A) and non-degenerate healthy (C57BL/6J; B) mice following quasi-subretinal or intravitreal delivery of LNPs (150 ng/µL) was carried out 24 h post transfection. Non-treated control (NTC) retinas served as controls. Each dot represents a single field of view (FoV), and colors indicate individual biological replicates (n = 3 per condition). One FoV corresponds to a single confocal micrograph (212.55 µm × 212.55 µm). For visualization purposes, the y-axis in panel A is truncated; two values from the subretinal condition exceed the displayed range and are not shown but were included in the statistical analysis. The subretinal dataset in panel A corresponds to the LNPb condition shown in Figure S3. Data in panel A were analyzed using the Mann–Whitney U test, whereas data in panel B were analyzed using one-way ANOVA followed by Tukey's HSD post hoc test. Unless otherwise indicated, data are presented as mean ± SEM. Statistical significance is denoted as ns (not significant), *$p < 0.05$, **$p < 0.01$, ***$p < 0.001$, and ****$p < 0.0001$.

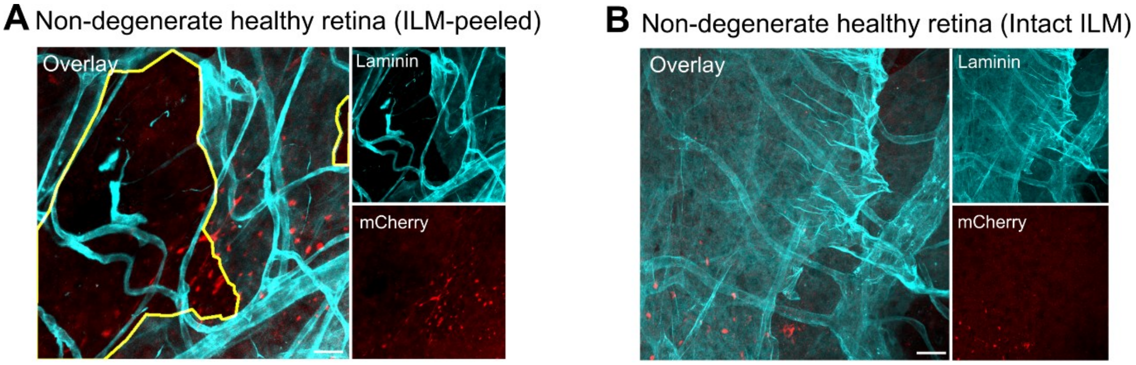

Figure S5: ILM-peeling promotes cellular uptake of cmRNA^{mcherry}m$^1\Psi_{1.0}$m^{5}C$_{1.0}$-LNP

ILM was partially removed during explantation of non-degenerate healthy explants (C57BL/6J). A representative confocal micrograph of (A) partially removed ILM and (B) intact ILM. Flat-mount retinal explants were double-stained with ILM marker laminin (cyan) and mCherry (red). ILM-peeled areas are highlighted (yellow). Scale bar: 20 µm.

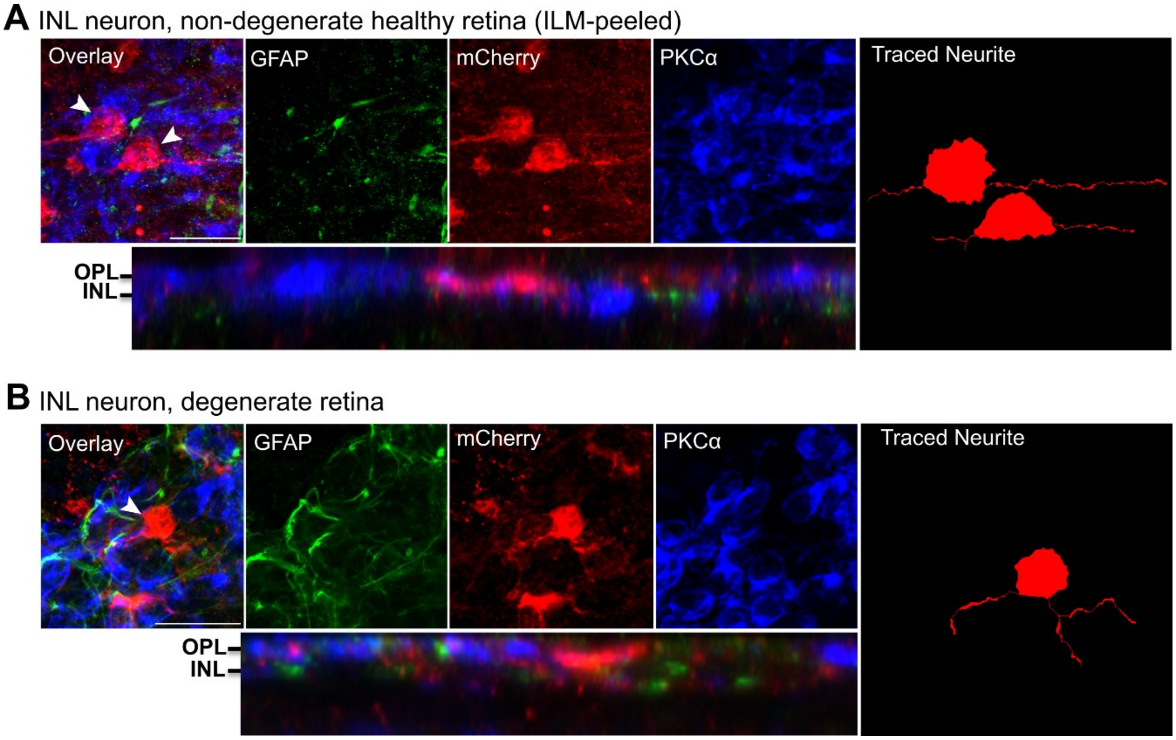

Figure S6: cmRNA^{mcherry}m$^{1}\Psi_{1.0}$m^{5}C$_{1.0}$-LNP transfects non-degenerate healthy and degenerate retinas.

Exemplary image from a high-resolution volume scan of (A) non-degenerate healthy explants (C57BL/6J) following intravitreal (ILM-peeled) and (B) degenerate explants (rd1, C3H) after subretinal administration. Flat-mount staining was performed using GFAP (green), mCherry (red) and PKCα (blue). Left panel: z-projection of the neuron-like structure with maximum intensity, with corresponding montage. Right panel: corresponding traced neurites. Arrowhead represents the associated traced neurite in the right panel. OPL: outer plexiform layer, INL: inner nuclear layer. Scale bar: 20 μm.

A

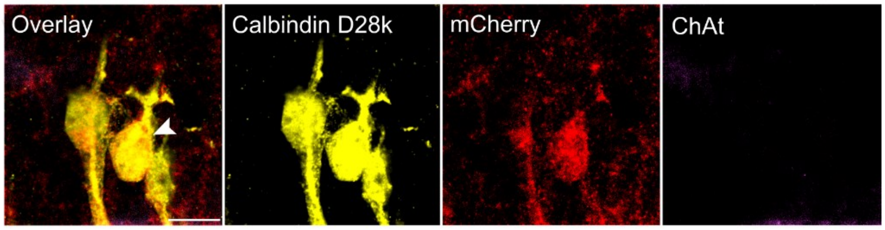

Figure S7: cmRNA^{mcherry}m$^1\Psi_{1.0}$m^{5}C$_{1.0}$-LNP transfects neuron.

Exemplary image from a high-resolution volume scan of (A) degenerate explants (rd1, C3H) following subretinal administration. Flat-mount staining was performed with Calbindin-D28K (yellow), mCherry (red) and ChAt (magenta). Arrow indicates overlap of mCherry and Calbindin-D28k within the soma. Scale bar: 10 µm.
