# Supplementary material for: Lipid nanoparticles enable mRNA delivery to diverse cell types of the inner Retina
Source: Mol Ther Nucleic Acids. 2026 Jun 30;37(3):102996. doi: 10.1016/j.omtn.2026.102996 (PMC13382181; doi:10.1016/j.omtn.2026.102996)
Supplement: Document S2. Article plus supplemental information [file mmc2.pdf]

Molecular Therapy
Nucleic Acids
Original Article

Lipid nanoparticles enable mRNA delivery to diverse cell types of the inner Retina

Sumit Biswas,[1] Eleonora Carpentiero,[1] Nermina Xhaferri,[1] Paula Streckenbach,[1] Vijay Renigunta,[2] and Moritz Lindner[1,3,4]

[1]Retinal Physiology and Gene Therapy Lab, Department of Neurophysiology, Institute of Physiology and Pathophysiology, University of Marburg, 35037 Marburg, Germany; [2]Department of Neurophysiology, Institute of Physiology and Pathophysiology, University of Marburg, 35037 Marburg, Germany; [3]The Nuffield Laboratory of Ophthalmology, Sleep and Circadian Neuroscience Institute, Nuffield Department of Clinical Neurosciences, University of Oxford, Oxford OX1 3QU, UK; [4]Department of Ophthalmology, University Hospitals of Giessen and Marburg, Marburg Campus, Marburg, Germany

Lipid nanoparticles (LNPs) have emerged as a promising platform for retinal genetic therapy, offering a non-viral alternative to adeno-associated viruses. Although LNPs can transfect outer retinal cells, their transfection profile across inner retinal cell types remains insufficiently characterized. Here, we systematically assessed the cell-type transfection profile of conventional LNPs encapsulating chemically modified mRNA encoding mCherry in murine retinal explants, complemented by experiments in dissociated retinal cell cultures. We compared quasi-subretinal and quasi-intravitreal administrations and evaluated how retinal degeneration and inner limiting membrane (ILM) integrity influence LNP-mediated transfections. We observed that LNPs efficiently transfected Müller glia under all experimental conditions. In addition, LNPs transfected several other retinal cell types, including neurons in dissociated cells and explants, and vascular cells exclusively in explants. Subretinal delivery resulted in higher transfection rates than intravitreal administration, and overall efficiency was higher in degenerate as compared to non-degenerate healthy retinas. In healthy retinas, removal of ILM increased transfection efficiency following intravitreal administration. Together, these findings demonstrate that conventional LNPs can transfect a broader range of retinal cell types than previously recognized and highlight LNPs as a tool for mRNA delivery to the retina, with applications in gene supplementation, editing, and regenerative therapies for inner retinal disorders.

INTRODUCTION

Over the last two decades the eye has emerged as a key target organ for gene therapy and numerous retinal disorders are currently the focus of preclinical and clinical development.[1,2] Voretigene neparvovec for Leber's congenital amaurosis and revakinagene taroretcel for macular telangiectasia type II have now received regulatory approval.[1,3] The immune-privileged status of the retina and the anatomical compartmentalization of the eye, the later supporting the achievement of high local vector concentrations, have both facilitated this rapid development.[4] Although Adeno-associated viruses (AAV) have become the preferred vector for retinal gene therapy, their limited packaging capacity, makes them unideal for delivering larger genes.[5] Moreover, AAV-mediated gene therapy results in persistent transgene expression, which is undesirable for certain applications like CRISPR-mediated gene editing.[6]

In recent years, lipid nanoparticle (LNP)-mediated RNA delivery has emerged as a novel variant of genetic therapy. This approach can be used when transient delivery of genetic material (mostly in form of mRNA) is desired, as exemplified by the approved mRNA-based vaccines against SARS-COV-2.[7] By delivering regulatory RNA, as in the approved Patisiran treatment for hereditary amyloidosis,[8] or mRNA encoding for gene editors, LNPs can be used acting on gene level and even as a form of classical gene therapy.[9] Moreover, the higher payload offered by LNPs makes them an ideal alternative in scenarios where the packaging capacity of AAV is insufficient.[10]

Until now, only few studies have addressed the use of LNP-mediated RNA transfer in the context of the retina and retinal disease.[11–16] Early investigations demonstrated that conventional lipid nanoparticles, depending on the route of delivery, either mediate transfection primarily of the retinal pigment epithelium (RPE) cells or Müller glia following subretinal or intravitreal delivery, respectively.[11,13] More recently, optimizations in LNP formulations enabled the transfection of RPE cells also by intravitreal delivery[11] and the design of peptide-guided LNPs as well as the introduction of certain lipid moieties have enabled efficient transfection of photoreceptors.[14,16,17] While most of these studies have been conducted in mice, there is now evidence that similar transfection patterns can be obtained in non-human primates and on cultured human donor retinas.[12,16]

Despite this progress, a comprehensive understanding of the cell-type transfection profile of LNPs, specifically within the inner retina, is missing. In particular, it remains unclear which retinal cell types,

Received 26 November 2025; accepted 29 June 2026;
https://doi.org/10.1016/j.omtn.2026.102996.

Correspondence: Moritz Lindner, Retinal Physiology and Gene Therapy Group, Department of Neurophysiology, Institute of Physiology and Pathophysiology, University of Marburg, 35037 Marburg, Germany.
E-mail: moritz@lindnerlab.de

Published by Elsevier Inc. on behalf of The American Society of Gene and Cell Therapy.
This is an open access article under the CC BY license (http://creativecommons.org/licenses/by/4.0/).

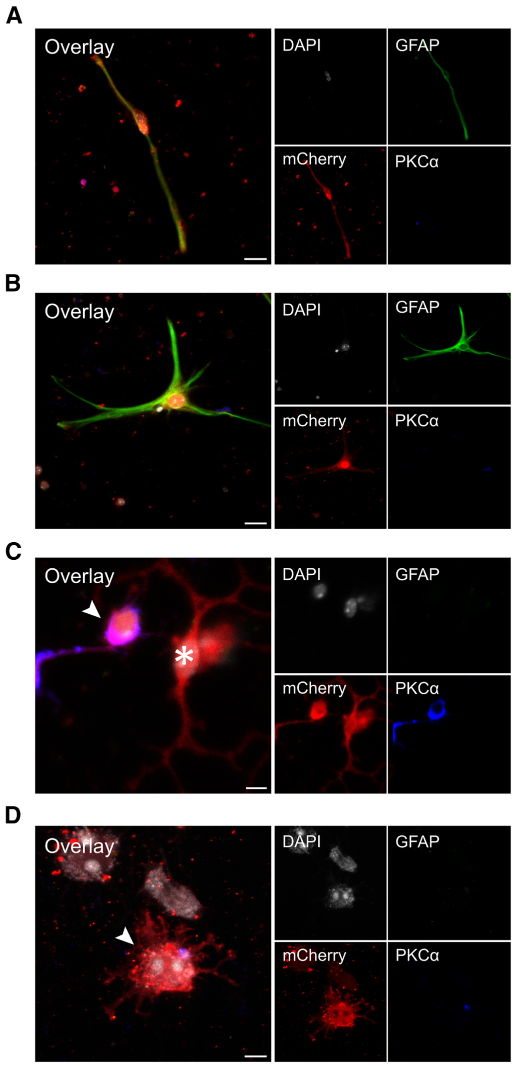

Figure 1. Transfection of cultured dissociated retinal cells using cmRNA^{mcherry}m$^1\Psi_{1.0}$m^{5}C$_{1.0}$-LNP

Representative confocal micrographs of dissociated primary retinal cells from rd1 retina-degenerate mice, fixed and immunostained 24 h post-transfection for mCherry (red), the Müller glia and astrocyte marker GFAP (green), and the rod bipolar cell marker PKCα (blue). Counterstain: DAPI (gray). (A) Müller glia. (B) Astrocyte. (C) Rod bipolar cell and other neuron. (D) Macrophage. Scale bars for (A), (B), and (D), 10 μm; (C), 5 μm.

besides Müller glia, can be transfected by conventional LNPs and how structural barriers such as the inner limiting membrane (ILM) and pathological changes associated with retinal degeneration modulate delivery efficiency. A better understanding of these aspects, however, would help to develop LNP-based treatments for a spectrum of retinal diseases, including optic neuropathies, congenital stationary night blindness, and age-related macular degeneration.

In this study, we systematically assess the cell-type transfection patterns of conventional LNPs in murine retinal explants. We evaluate the influence of delivery route, ILM integrity, and retinal degeneration on LNP-mediated transfection efficacy, and demonstrate that LNP can transfect a broader spectrum of retinal cell types than previously thought. Specifically, we observe that they are capable of transfecting inner retinal cell types beyond phagocytosing cells, including vasculature as well as neurons. These findings will support further development of gene therapies for inner retinal disease or conditions where production of soluble gene products is required.

RESULTS

As previous studies had mainly reported transfection of Müller glia in the inner retina, we were particularly interested in investigating the transfection profile of LNPs beyond Müller cells. In order to assess if retinal cell types can be transfected using LNPs at all, we commenced this investigation in cultured dissociated retinal cells. By this approach, LNPs would have direct access to all cell types of the inner retina[18] and thus allowing us to assess whether non-glial retinal cell types can support LNP-mediated mRNA expression beyond the influence of any anatomical barriers. We therefore transfected the cultured dissociated retinal cells with cmRNA^{mCherry}m$^1\Psi_{1.0}$-m^{5}C$_{1.0}$–LNPs and 24 h later, mCherry expression was evaluated. Many transfected cells are immunopositive for glial fibrillary acidic protein (GFAP) and had a drawn-out structure typical for Müller cells (Figure 1A). Among the GFAP$^+$ cells, we also encountered some that were rather star-shaped, thus likely representing astrocytes (Figure 1B). Most interestingly, among the transfected cells we also repeatedly found GFAP immunonegative cells with a neuron-like morphology (Figure 1C, asterisk), whereof one specifically had a typical bipolar-cell morphology and was additionally immunopositive for the ON-Bipolar cell marker protein kinase C alpha (PKCα) (Figure 1C, arrowhead). The rest of these neuron-shaped cells were immunonegative for PKCα (and all other markers tested). Some mCherry$^+$ cells also exhibited morphological similarities with macrophages (Figure 1D, arrowhead). Thus, at least in the absence of anatomical barriers, LNPs are able to transfect diverse retinal cell types, including neurons. Notably, as we did not quantify, by these experiments no conclusion on a preferred or relative tropism of the employed LNP can be made.

We next explored which cell types could be transfected by LNPs in the intact retina. As a starting point, we applied cmRNA^{mCherry}m$^1\Psi_{1.0}$-m^{5}C$_{1.0}$–LNPs to retinal explants from retina-degenerate rd1 mice that were cultured ganglion cell-side down. Thus, application of the LNPs to the free surface of the explant, i.e., the side directed away from the membrane, would represent a quasi-subretinal route of delivery. Transfection was performed immediately after taking the explants into culture (hereafter referred to as acute transfection). Twenty-four hours later the retinas were fixed and assessed for mCherry immunofluorescence. Robust mCherry expression could be observed throughout all treated explants (Figure 2A). On average, we observed 34.89 [±4.01] mCherry$^+$ cells per field of view (FoV) (Figure 2C) which equaled an overall transfection efficiency of 14.12% [±1.62]. Notably, there was a substantial variability in transfection yield within individual retinas

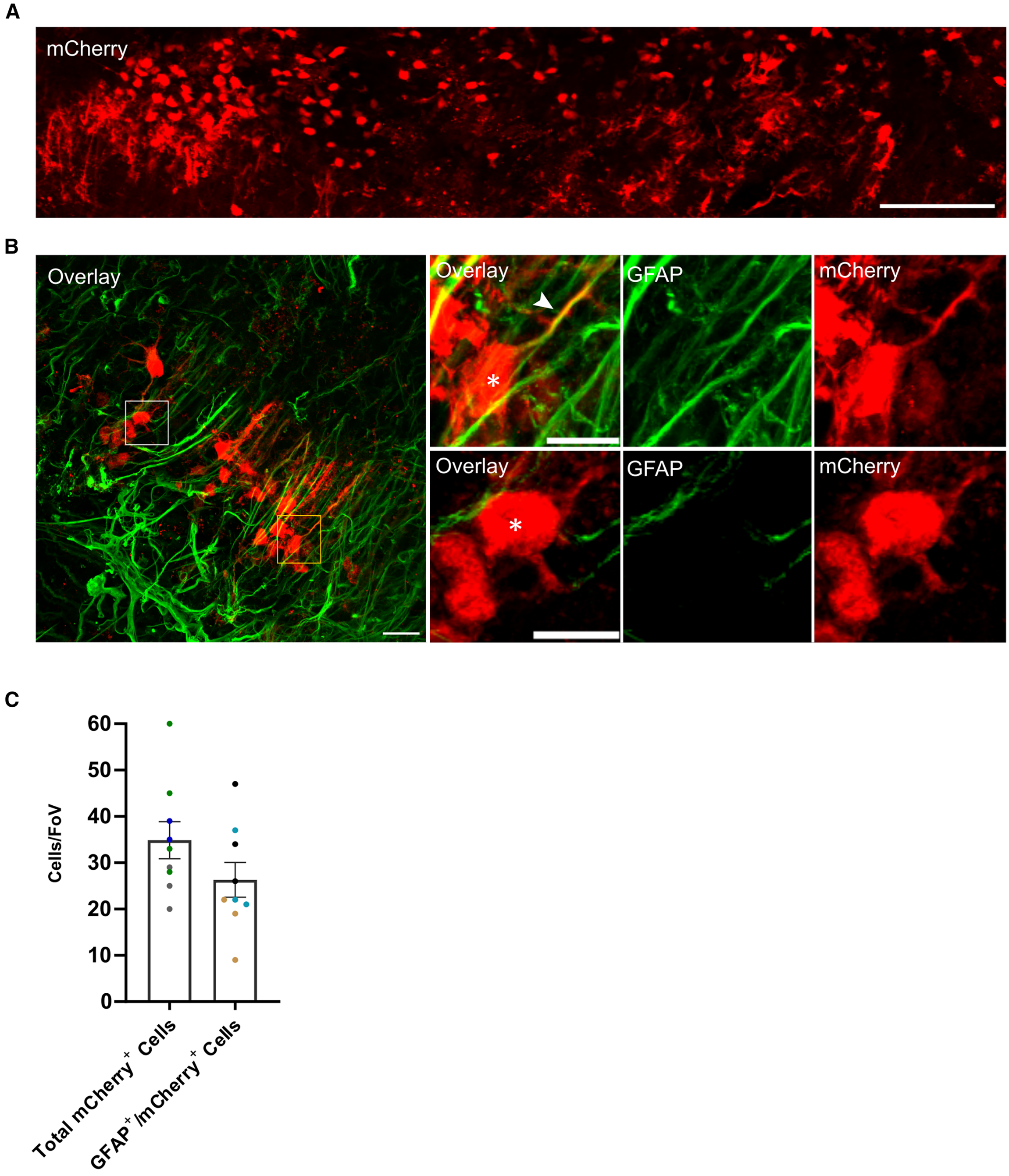

Figure 2. Transfection pattern in retinal explants from retina-degenerate eyes using cmRNA^{mCherry}m$^{1}\Psi_{1.0}$m^{5}C$_{1.0}$-LNP

Retinal explants from rd1 retina-degenerate mice transfected by quasi-subretinal delivery immediately after explantation (150 ng/μL). (A) Stitched composite image was generated from multiple overlapping micrographs to visualize the full extent of transfection across the explant. (B) Exemplary micrographs of explants immunostained for mCherry (red) and GFAP (green). (C) Number of total mCherry^{+} and GFAP^{+}/mCherry^{+} cells per field of view (FoV) assessed 24 h post-transfection. Note that GFAP does not stain the somata of Müller glia but rather their processes, hence, cells were classified as GFAP^{+} based on their processes. Dots represent data acquired from an individual FoV, colors represent individual biological replicates (3 per condition). Boxes and error bars represent mean and SEM, respectively. A FoV represents a single confocal micrograph (212.55 μm × 212.55 μm). Scale bars for (A), 150 μm; (B), 20 μm, (B) upper and lower images, 10 μm.

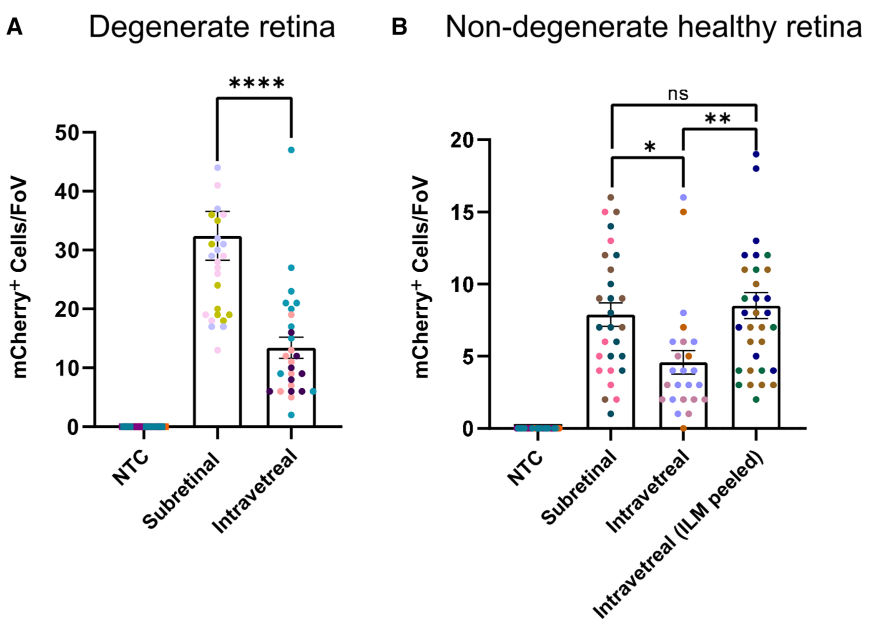

Figure 3. Transfection yield in degenerate and non-degenerate healthy retinal explants

Quantification of mCherry$^+$ cells in retinal explants from degenerate (rd1, C3H; A) and non-degenerate healthy (C57BL/6J; B) mice following quasi-subretinal or intravitreal delivery of LNPs (150 ng/μL). Non-treated control (NTC) retinas were used as controls. Dots represent data acquired from an individual FoV, colors represent individual biological replicates (3 per condition). A FoV represents a single confocal micrograph (212.55 μm × 212.55 μm). To enhance visualization in (A), the *y* axis was truncated. Two outlier values from subretinal administration fall beyond the axis limit and are not displayed but were still included in the statistical analysis. Data in (A) were analyzed using the Mann-Whitney test, and data in (B) were analyzed using one-way ANOVA followed by Tukey's HSD post-hoc test. Data are presented as mean ± SEM. Statistical significance is indicated as ns (non-significant), *$p < 0.05$, **$p < 0.01$, ***$p < 0.001$ or ****$p < 0.0001$.

(Figure S1), which is consistent with previous observations.[12] To find out how many of the transfected cells would be Müller glia, we assessed co-incidence of the mCherry signal with GFAP immunoreactivity within an individual cell (Figure 2B, upper panel, arrowhead indicating the overlap of mCherry$^+$ cell with Müller glia process; asterisk pointing to the corresponding soma). Indeed, 26.33 [±3.76] cells/FOV were GFAP$^+$/mCherry$^+$ (Figure 2C) while the others showed no evidence of GFAP signal neither in their soma nor their extensions (Figure 2B, lower panel, asterisk).

To confirm that our observations were not biased by potential acute cell stress artifacts induced by the explantation process, we repeated these assessments in retinas kept in culture for two days before transfection (denoted as late transfection, Figures S2A–S2C). Except for a slightly, albeit statistically significant, lower proportion of GFAP$^-$ cells (late: 5.88 [±0.64], acute: 8.56 [±0.88] GFAP$^-$/mCherry$^+$ cells/FOV; $p < 0.05$, Figure S2D) transfection yield was largely similar. Therefore, in all subsequent experiments, we transfected the retinas immediately after explantation.

We next moved on to analyze the impact of the route of LNP delivery (intravitreal vs. subretinal) as well as potential differences in transfection yield between degenerate and non-degenerate healthy retinas. By far the largest number of mCherry-positive cells was observed after quasi-subretinal administration in degenerate explants. With 32.44 [±4.16] cells/FOV and a transfection efficiency of 13.13% [±1.68] this was significantly higher than after intravitreal administrations (13.44 [±1.79] cells/FOV, $p < 0.0001$; Figure 3A and 5.44% [±0.72] transfection efficiency; Figure S4A). A similar trend was observed in healthy explants, though transfection yields were overall lower. Subretinal delivery again outperformed intravitreal delivery both in terms of transfected cell density (intravitreal: 4.58 [±0.80], subretinal: 7.90 [±0.82] cells/FOV; $p < 0.05$; Figure 3B) and transfection efficiency (intravitreal: 1.04% [±0.18], subretinal: 1.71% [±0.18], Figure S4B). Given the reduced transfection in healthy retina and the known structural differences in the ILM between healthy and degenerate tissue,[19,20] we hypothesized that an intact ILM might hinder intravitreal transfection upon intravitreal delivery. Therefore, we performed an additional experiment where we mechanically removed the ILM from non-degenerate healthy explants (Figure S5). Indeed, after ILM peeling, quasi-intravitreal LNP administration yielded 8.52 [±0.92] mCherry$^+$cells/FOV and a transfection efficiency of 1.85% [±0.20], which is significantly more than what was achieved without peeling ($p < 0.01$; Figures 3B and S4).

To characterize the non-glial targets in the intact retina more in detail, we performed an in-depth morphological analysis of the GFAP$^-$ mCherry$^+$ cells in the cultured explants. Following quasi-intravitreal delivery, we observed mCherry expression in structures resembling blood vessels, thus in cells representing pericytes or endothelial cells (Figure 4). Similar observations could not be made following quasi-subretinal delivery. Moreover, consistent with our observations in dissociated retinal cells, also in the intact retina, we could observe mCherry$^+$ cells with neuron-like morphology. We further assessed these by neurite tracing on confocal volume scans. We found that the somata of these cells were typically located to the outer portion of the inner nuclear layer (INL) and were consistently immunonegative for PKCα. By localization and morphology, in particular, the horizontal orientation of their neurites, we postulate that these are horizontal cells (Figures 5A, 5B, and S6A, and S6B). To further support this hypothesis, we assessed the co-localization of mCherry fluorescence with Calbindin, an established marker for horizontal cells (Figure S7, arrowhead indicating the overlapping immunoreactivity of

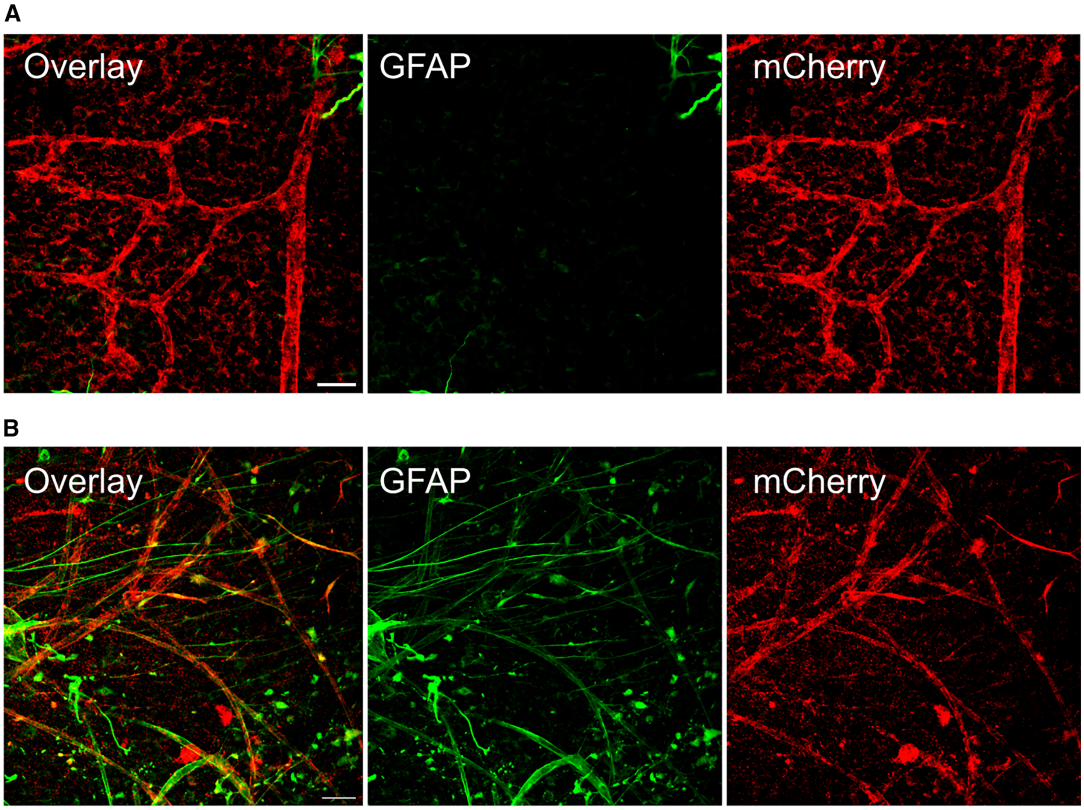

Figure 4. cmRNA^{mCherry}m$^1\Psi_{1.0}$m^{5}C$_{1.0}$-LNP transfects blood vessels

Micrographs acquired from non-degenerate healthy retinal explants from C57BL/6J mice that were subjected to quasi-intravitreal delivery of LNPs (150 ng/μL). Immunostaining for mCherry (red) showed the transfection of vessel-like structures in (A) non-degenerate healthy retina (ILM-peeled) and (B) in non-degenerate healthy retina after quasi-intravitreal administration. Note that occasionally one can observe GFAP-immunoreactivity (green) closely aligning (but not completely overlapping) with these structures (presumably representing astrocytes sheathing those vessels). Scale bars: 20 μm. For better visualization background noise was removed using despeckling and local averaging (median) filters. Data obtained from 3 independent biological replicates. Similar observations were made on degenerate rd1 retinas following quasi-intravitreal delivery (not shown).

mCherry$^+$ and horizontal cell within an individual cell soma). Additionally in degenerate retinas, we also detected a small number of mCherry$^+$ cells distributed within the ganglion cell layer (GCL) of the retina (Figure 5C).

While LNPs have been generally reported to exert little toxicity, LNPs themselves or the genetic material they deliver may trigger inflammatory or other stress responses in the retina.[17,21] A sensitive marker for retinal stress is Müller glia activation, which is associated with an increase in GFAP expression in these cells.[22] We therefore assessed differences in the levels of GFAP immunoreactivity. In degenerate explants, GFAP levels were markedly and significantly increased following intravitreal administration ($p < 0.0001$) as well as subretinal administration ($p < 0.01$) compared to non-transfected control (NTC) explants (Figures 6A and 6B; Table S1). In non-degenerate healthy explants, intravitreal administration without prior ILM peeling only led to a small and non-significant ($p < 0.507$) increase in GFAP signal as compared to NTC controls. Upon subretinal administration, by comparison, this increase was robust and statistically significant ($p < 0.001$), yet still substantially lower than what had been observed in the degenerate retinas. In ILM-peeled healthy explants following intravitreal administration, GFAP signal was substantially higher than in retinas without prior peeling ($p < 0.01$) (Figures 6C and 6D; Table S1).

DISCUSSION

In the present study, we systematically characterized the cell-type transfection profile of lipid nanoparticles delivering chemically modified mRNA in murine retinal explants. We found that conventional LNPs transfect a broader range of retinal cell types than previously recognized including neurons and vascular cells. Quantitative analysis in explants revealed that quasi-subretinal delivery was significantly more efficient than quasi-intravitreal administration, although removal of the inner limiting membrane markedly enhanced intravitreal delivery efficacy. Furthermore, we observed that overall transfection efficiency was higher in degenerate rd1 retinas than in non-degenerate controls.

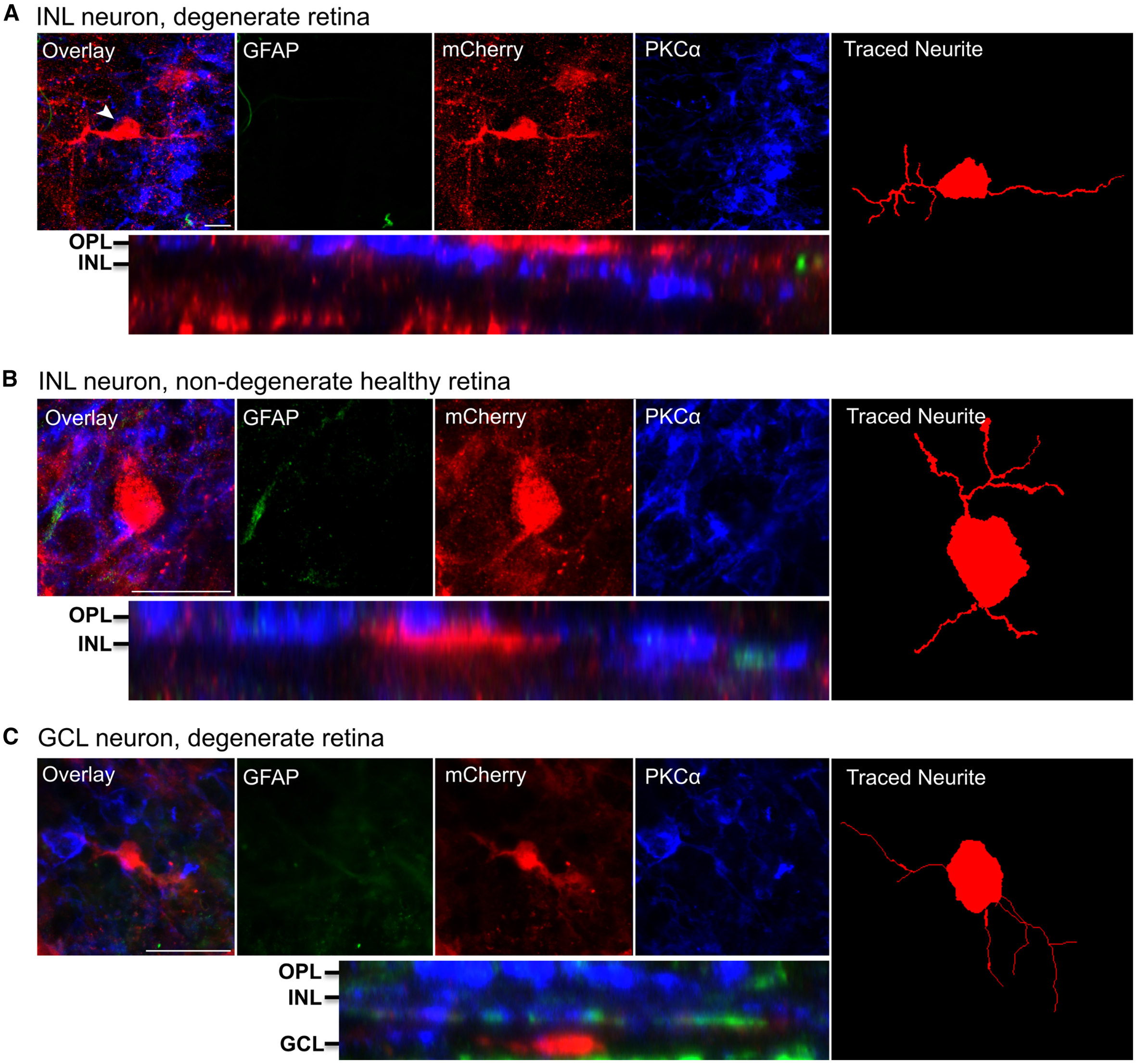

Figure 5. cmRNA^{mCherry}m$^1\Psi_{1.0}$m^{5}C$_{1.0}$-LNP transfects retinal neurons

Exemplary high-resolution volume scans from degenerate (rd1, C3H; A and C) retinal explants 24 h post intravitreal and subretinal transfection, respectively and non-degenerate healthy (C57BL/6J; B) retinal explants 24 h following subretinal transfection. Retinal explants were triple-labeled against mCherry (red), GFAP (green) and PKCα (blue). Corresponding orthogonal representations of the cells are shown below. Arrowhead represents the associated traced neurite in the right panel. OPL: outer plexiform layer, INL: inner nuclear layer, GCL: ganglion cell layer. Scale bars for (A) and (B), 20 μm.

To analyze the effect of the route of delivery on LNP transfection efficacy we utilized murine retinal explants as a model system. Culturing them with the ganglion cell side attached to the cell culture insert enabled a quasi-subretinal route for LNP delivery while the reverse orientation (i.e., outer retina toward culture insert) allowed for quasi-intravitreal LNP delivery. We found a substantially higher transfection yield with subretinal delivery as compared to intravitreal delivery. Few studies have previously assessed the ability of LNPs to transfect inner retinal neurons using either an intravitreal[12] or subretinal route of delivery.[13,16] Yet, direct comparisons between the two approaches are limited[11,15] and widely descriptive. While most authors reported at least some degree of inner retinal cell (specifically Müller glia) transfection regardless of the delivery route chosen, Ryals et al. explicitly state that they did not observe any inner retinal transfection upon subretinal LNP delivery.[11] In this regard, the quantitative data we provide here are helpful in setting an objective basis for future analysis and it is particularly relevant to note that in our experience, subretinal delivery has been substantially more efficient than intravitreal delivery, despite similar local LNP concentrations, unless the ILM was removed. It is of importance to note that

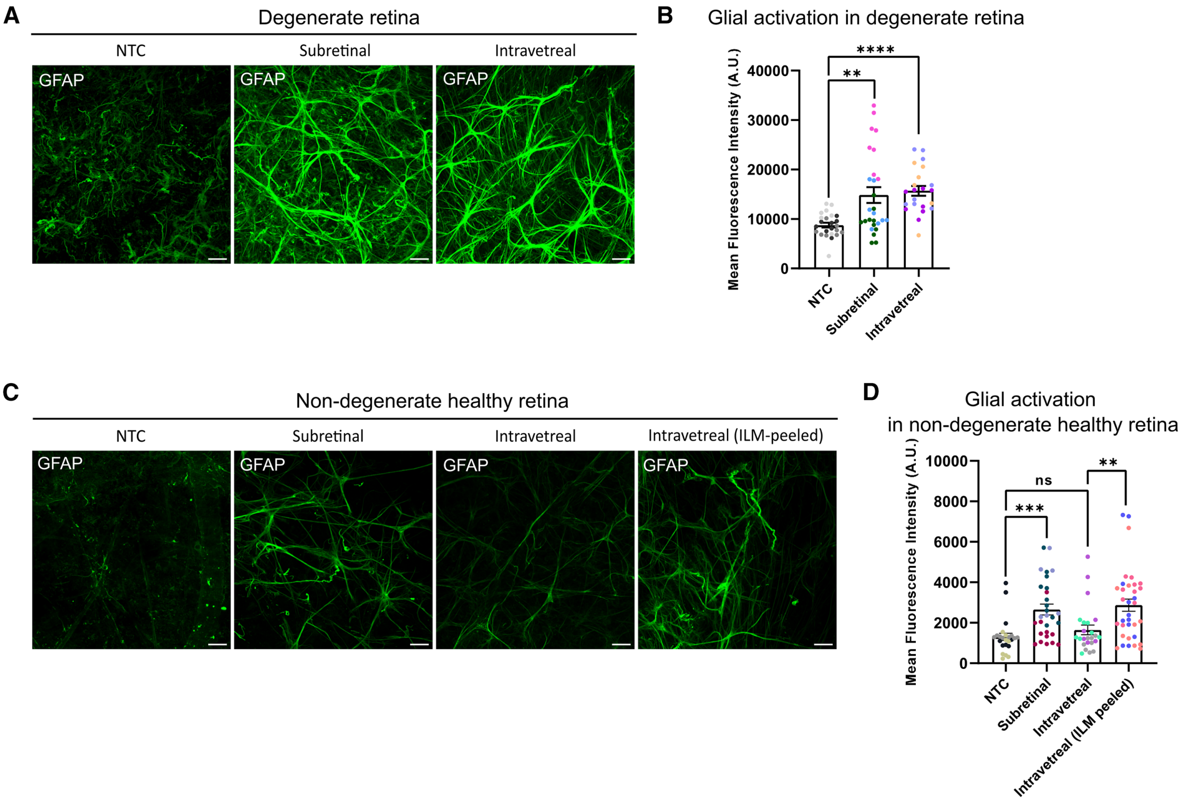

Figure 6. cmRNA^{mCherry}m$^1\Psi_{1.0}$m^{5}C$_{1.0}$-LNP mediated glial activation across retinal explants

Representative confocal micrographs of GFAP immunoreactivity from (A) degenerate (rd1, C3H) and (C) non-degenerate healthy (C57BL/6J) explants after 24 h post transfection following quasi-intravitreal and quasi-subretinal administration. Quantification of GFAP signal intensity (mean fluorescence intensity, MFI in arbitrary units [A.U.]) in (B) degenerate and (D) non-degenerate healthy retina. Respective non-treated control (NTC) groups were used as controls. Scale bars: 20 μm. Dots represent data acquired from an individual FoV, colors represent individual biological replicates (3 per condition). A FoV represents a single confocal micrograph (212.55 μm × 212.55 μm). Statistical significance in (B) and (D) was assessed using one-way ANOVA followed by Dunnett's test. In (D), comparisons between intravitreal administration in retinas and intravitreal administration in ILM-peeled retinas were analyzed using the Mann-Whitney test. Data are expressed as mean ± SEM. *p* values are reported as ns (non-significant), *$p < 0.05$, **$p < 0.01$, ***$p < 0.001$ or ****$p < 0.0001$.

despite the production of two batches of LNPs (LNPa and LNPb), these batches of LNPs provided a similar transfection efficiency in degenerate retinas following subretinal administration (Figure S3). This consistency indicates that batch-to-batch variability did not significantly impact functional delivery in this context. Although these findings may suggest that the observed effects are ultimately governed by specific LNP physicochemical properties rather than the particle size itself, these parameters were not systematically assessed in the present study. Therefore, the contribution of particle size and its interplay with other physicochemical properties in transfecting neuronal retina warrants for rigorous investigation in future.

Notably, in this study, we used a relatively standard LNP formulation not intended to target any cellular population in specific. Instead, the primary aim was to establish a foundational understanding of which retinal cell populations can, in principle, be accessed by conventional mRNA-LNP systems under different anatomical and pathological conditions. Current LNP technologies offer substantially broader design possibilities, including advanced ionizable lipids and cell-specific targeting strategies. Our findings provide an important framework for the future development of peptide-guided or otherwise engineered LNP formulations with enhanced selectivity toward the retinal target cell population of choice.

The frequent transfection of Müller glial cells by mRNA-LNP complexes observed in this work in cultured retinal explants is in agreement with observations reported in *ex vivo* and *in vivo* mouse models across different mRNA-LNP formulations.[11–13,17,21,23,24] Nevertheless, it is important to keep in mind that experiments in this study were exclusively conducted *ex vivo*. While the model system of retinal explant culture and the quasi-intravitreal/quasi-subretinal delivery strategy is well established,[25–28] it does not fully recapitulate native anatomical environment in of the retina. For instance, when thinking in an intravitreal route of delivery, the viscosity and composition of the vitreous may affect LNP behavior, for instance by formation of a protein corona,[29,30] and thereby affect transfection

efficiency. This aspect warrants more systematic investigation in future studies.

The reasons for the higher transfection yield we observed upon subretinal as compared to intravitreal LNP delivery are overall unclear. We hypothesize that the ILM imposes a structural barrier due to both, pore size and its negative charge, obstructing particle transmission.[31] This concept is supported by our observation that transfection efficiency of intravitreally applied LNPs improved after mechanical ILM removal. Clearly, from the subretinal side, the outer limiting membrane (OLM) imposes a somewhat similar diffusion barrier. Yet, as opposed to the actual basement membrane nature of the ILM, the OLM is made up of the tight junctions between photoreceptors and Müller cell feet.[32] Thus, while the OLM imposes a diffusion barrier, it would not necessarily hinder particle uptake into Müller cells. In fact, there are several reported examples of material uptake into Müller cells from their apical site.[33,34] It should be noted that in our cultured explants, RPE cells were mostly absent. In an intact, non-degenerate healthy eye, these cells heavily phagocytose and may therefore scavenge LNPs that could otherwise transfect cells of the neuroretina. This impact of RPE cells might well explain why no inner retinal transfection was observed with subretinal LNP delivery in previous studies.[11]

In practice, subretinal delivery may be preferred when mRNA therapy would be a one-off treatment, as this allows for easy achievement of high local LNP concentrations, while the intravitreal route is less invasive and also possible in outpatient settings. In this regard, alternative methods for ILM removal, like enzymatic digestion[35] or photochemical destruction[36] may prove helpful to increase transfection yields.

We also observed substantially higher transfection efficiency in explants from retina degenerate (rd1) mice; an observation that was particularly pronounced upon subretinal delivery (Figures 3 and S4). This finding is of relevance given that most applications of LNP-mediated mRNA delivery to the retina are in diseased retinas. Clearly, the course of retinal degeneration is rapid in the rd1 mouse, and it is unclear to what extent our observations can be generalized to other retinal diseases. However, changes to the limiting membranes, in particular the OLM, are observed relatively early in the disease course and resemble those seen in other types of retinal degeneration.[37] In this regard, the rd1 model that we have studied herein represents an informative starting point for further studies on more specific disease models.

Previous studies have identified Müller glia as the primary inner retinal cell type transfected by this standard LNP as well as any other LNP formulations.[11,13,16,17] Even where similarly formulated LNPs were used, transfection of other inner retinal neurons was not reported. Only Chambers et al. additionally observed the transfection of macrophages.[12] The reasons for this apparent discrepancy are not yet entirely clear. We speculate that by analyzing confocal volume scans covering large areas from retinal flat-mounts, we were able to obtain a more detailed view on the diversity of retinal cell types transfected by LNPs. Herein, we have explored the capacity of LNPs to transfect inner retinal cells beyond these classically phagocytosing cells. On cultured dissociated retinal cells, where the anatomical barriers of the intact retina are absent, we observed that not only Müller glia, and macrophages are indeed targets of LNPs, but also astrocytes and neurons can be transfected. It is worth mentioning that we did not confirm the presence of each retinal cell type in our dissociated culture. While it is clear that all cell types survive the dissociation process[18] and even neuronal populations can be maintained over several day,[38] we cannot fully exclude that individual cell types were underrepresented as compared to the native situation.

Subsequent experiments on cultured intact retinas overall confirmed this observation in a more native setting and additionally showed that also endothelial cells and/or pericytes were among those transfected. However, we did not systematically assess the relative transfection rates for the individual cell types. In the intact retina, however, we found that the transfected cells are $GFAP^{+}$ (i.e., Müller Glia or astrocytes), by visual assessment, the vast majority of these being Müller cells. Interestingly, among the remaining GFAP immunonegative cells, both neurons and vascular cells were observed and thus reveal to be feasible targets for LNPs. Hence, tailoring LNP formulations to the intended target cell type could facilitate the development of mRNA therapeutics with precise cell-type specificity. The necessary modifications for both, improved endothelial and neuronal targeting have been studied extensively outside the eye[39–41] and could be adopted for ocular delivery.

Although by characterizing distinctive morphological features and using immunohistochemical markers we were able to coarsely infer cell identity, a more fine-granular classification of the transfected cell types, particularly among neurons, would be desirable. Future studies employing single-cell RNA sequencing, a broader set of immunohistochemical markers on retinal cryosections or multicolor flow cytometry will be helpful to comprehensively map the tropism of LNPs within the retinal cellular landscape.

Reactive Müller cell gliosis is a universal response mechanism of the retina to stress and activated Müller glia characteristically exhibit increased GFAP immunoreactivity.[22] We have therefore used GFAP signal intensity as a non-specific but sensitive marker with which to measure retinal stress in response to LNP administration. Indeed, we observed Müller cell activation following LNP administration for most conditions studied. Only after intravitreal LNP delivery to non-degenerate healthy retinas, no significant increase in GFAP signal could be observed. Since ILM-peeled explants exhibited a statistically significant increase in GFAP signal compared to non-peeled explants following intravitreal delivery of LNPs, it is possible that the ILM protects the retina from LNP-induced stress while also representing a relative obstacle for transfection. It is unclear, whether this effect is simply related to the lower intraretinal effective dose of LNPs or if there is any sort of additional protective effect (e.g., by

charge filtering). In this context, previous studies have focused on assessing immune activation in response to LNP delivery and find no evidence for immune activation upon intravitreal delivery[12,17,21] and some upon subretinal delivery.[16,17] Of note, Müller glia activation may also occur independent of immune activation and thus represents a complementary way of assessing the retina response to LNP-mediated transfection. Nevertheless, assessing GFAP as retinal stress marker does no means assess cellular or organ stress in its full complexity. A more differentiated assessment, e.g., by qPCR or established ELISA assays is necessary and should include immune profiling, e.g., as implemented in the established RNA ImmunoGenic assay.[42] Minimizing the immunogenicity of the delivered RNA cargo could be achieved by further chemical nucleoside modifications, codon optimization, and/or incorporation of miR-142-3p binding sites into the 3′ UTR to restrict expression in immunogenic cell types.[7,43,44]

From a translational perspective, the key observation made in this study is that inner retinal cell types beyond the phagocytosing cells—including neurons—can be transfected using LNPs. In particular, the transfection of neurons is of relevance as it indicates that treatment of hereditary retinal disorders that have their primary cause in the inner retina, like several forms of congenital stationary night blindness (CSNB), by LNP-mediated mRNA delivery is generally feasible. Bipolar cells, the main therapeutic target in many CSNB subtypes, have proven difficult to transduce with AAV vectors.[45] LNPs would therefore provide an attractive alternative. Moreover, the 4.7 kb packaging ceiling of AAVs (promoter included) precludes delivery of large genes like TRPM1—a major CSNB locus—or of the multi-component gene-editing systems required for *in situ* correction.[6,46] LNPs, by contrast, have a much higher payload and can encapsulate all gene-editing components in a single particle. With regard to gene editing, the non-perpetual nature of LNP-mediated transfection is an additional advantage to minimize the risk of off-target effect.[6] Certainly, this will require further tweaking of LNP tropism in favor of neuronal transfection—wherefor recent advances made in transfecting brain neurons could serve as template.[41] In this regard, it is worth noting that in our studies on dissociated retinal cells transfected ($PKC\alpha^+$) ON-bipolar cells were particularly rare, suggesting that there might be cell-type specific challenges that need to be addressed.

Beyond these requirements for cell-type specific transfection, our results demonstrate that efficient transfection of Müller cells can be achieved using conventional LNPs with components that are all already employed in clinical settings[47] and an intravitreal route of delivery (enabling repeated administration e.g., in an outpatient setting), efficient transfection of at least of Müller cells can be achieved. Thus, this approach could prospectively be exploited in regenerative applications, where the aim is to reprogram Müller glia into neurons[48,49] or where soluble gene products are needed. Such situations include the production of VEGF-receptor fragments to treat exudative retinal disease,[50] complement inhibitors to halt atrophic AMD[1] or neurotrophic factors for glaucoma or macular telangiectasia type II.[51,52] In this context, pharmacokinetics could be adjusted using depot systems and materials[53] or self-amplifying mRNA formats.[54]

In addition to these translational considerations, our finding that LNPs can be efficiently used for gene delivery to cultured retinal explants is of practical relevance in laboratory research settings. Compared to alternative virus-mediated gene delivery, LNP-mediated expression occurs much faster, accelerating experiments and reducing the time it takes for cultured organs to drift.

Conclusions

Our findings demonstrate that conventional LNPs can successfully transfect a range of retinal cell types beyond Müller glia, including neurons and vascular cells, particularly in degenerate retinal tissue. Subretinal delivery consistently outperforms intravitreal administration in terms of transfection efficiency, although removal of the inner limiting membrane can partially overcome this limitation. These results highlight the potential of LNPs as a flexible and scalable platform for genetic therapies targeting inner retinal diseases, especially where transient expression or large payloads are required.

MATERIALS AND METHODS

mRNA and LNP formulation

mRNA encoding the fluorescent protein mCherry encapsulated in lipid nanoparticles were purchased from VectorBuilder (Chicago, USA). To generate $\text{cmRNA}^{mCherry}\text{m}^1\Psi_{1.0}\text{m}^5\text{C}_{1.0}$, uridine-tri-phosphate (UTP) and cytidine-tri-phosphate (CTP) were completely substituted by N^1-methylpseudouridine ($m^1\Psi$) and 5-methylcytosine (m^5C), respectively. LNP was formulated with (6Z,9Z,28Z,31Z)-Heptatriaconta-6,9,28,31-tetraen-19-yl 4-(dimethylamino)butanoate (Dlin-MC3-DMA), 1,2-Distearoyl-sn-glycero-3-phosphocholine (DSPC), cholesterol, and 1,2-dimyristoyl-rac-glycero-3-methoxypolyethylene glycol-2000 ($DMG\text{-}PEG_{2000}$) in a ratio of 50:10:38.5:1.5. The ratio of ionizable lipid nitrogen to mRNA phosphate (N/P) was 6. Subsequent quality assurance assays were performed by the manufacturer.

Two batches of LNP were formulated via NanoAssemblr (Precision NanoSystems, Canada) and characterized by Zetasizer Ultra (Malvern, USA) for the hydrodynamic diameter, polydispersity index (PDI) and zeta potential. Particle sizes were 73.7 nm and 101.1 nm, with polydispersity indices of 0.072 and 0.070, respectively. Zeta potentials were −4.07 mV and −9.82 mV, respectively.

Animals

C57BL/6J and C3H/HeOuJ mice were purchased from Charles River (Sulzfeld, Germany). Animal work was performed with approval of the relevant authorities and in accordance with the institutional Ethics Guidelines of Animal Care. All the mice were housed in a specific pathogen-free animal facility on a 12 h light-dark cycle with food and water available *ad libitum*. Tissue collection was carried out in accordance with federal law and institutional guidelines. For tissue collection, mice were decapitated following deep isoflurane (Baxter, Deerfield, Illinois, United States) anesthesia and retinas

were extracted and immediately transferred to CO_2-independent medium (Thermo Fisher Scientific, Waltham, MA, United States) as previously described.[55]

Primary retinal cell culture and transfection

Retinas were dissociated using the Papain Dissociation System (Worthington Biochemical Corporation, Lakewood, NJ, USA) with 250 μL of papain, 150 μL of ovomucoid, and 15 μL of DNase solution per retina. Centrifugation was performed at 124 × *g* for 5 min to separate cells from debris. Retinal cells were then transferred into culture medium (Neurobasal-A, Thermo Fisher Scientific supplemented with: 10% Dulbecco's Modified Eagle Medium, Thermo Fisher Scientific; 2.5% B-27 plus supplement, Thermo Fisher Scientific; 5 μg/mL forskolin, Sigma-Aldrich, St. Louis, USA; 10 ng/mL human ciliary neurotrophic factor, Miltenyi Biotec, Bergisch Gladbach, Germany) and seeded on Poly-D-Lysine and Laminin coated coverslip (Corning, New York, USA). Cells were transfected with cmRNA^{mCherry}m$^1\Psi_{1.0}$-m^{5}C$_{1.0}$–LNP at a final concentration of 0.7 ng/μL. 24 h after transfection, cells were fixed with 4% paraformaldehyde (PFA, Thermo Fisher Scientific) and stored in PBS at 4°C until further use.

Retinal explant preparation and transfection

Retinal explants were prepared following the method by Devoldere et al. with minor modifications.[15] After extraction, retinas were transferred onto semi-permeable PTFE membrane cell culture inserts (Merck, New Jersey, USA) either with photoreceptor side or the retinal ganglion cell side facing upwards and fixed to the insert by applying negative pressure. These two orientations of the explant with either the ganglion cell or photoreceptor side facing upwards would allow application of LNPs to that specific side of the retina by pipetting, thereby mimicking an intravitreal (ganglion cell side up) or subretinal (photoreceptor side up) route of delivery, respectively. This concept has been previously established by Pang et al.[25] We use the term "quasi" to refer to either of these routes and acknowledge that this *ex vivo* model does not fully recapitulate the native anatomic embedding of the retina.

Following establishment of the explant culture system, experiments were performed as described below. Culturing medium (Neurobasal-A supplemented with 1% B-27, 1% penicillin-streptomycin, 0.5% L-glutamine, 2.5% fetal bovine serum) was added below the inserts, after which the explants were cultured at 37°C. The explants were transfected either immediately after being taken into culture or after culturing for two days using cmRNA^{mCherry}m$^1\Psi_{1.0}$-m^{5}C$_{1.0}$–LNP at a final concentration of 150 ng/μL. 24 h post-transfection, retinal explants were fixed with 4% PFA and stored at 4°C until immunostaining.

Removal of the ILM

Before transferal onto the cell culture inserts, the ILM was stained using 0.4% trypan blue (Thermo Fisher Scientific) for 1–2 min. Excess trypan blue was removed by gently shaking the retina inside the preparation dish. Fine forceps (Fine Science Tools, Heidelberg, Germany) were used to remove the ILM under a dissecting microscope.

Immunocytochemistry and immunohistochemistry

Dissociated retinal cells were permeabilized with 0.1% Triton-X in PBS (0.1% PBSTX) and blocked with 5% goat serum. Primary antibody incubation was performed in 2.5% goat serum in PBS for 2 h and secondary antibody incubation for 1 h. All steps were carried out at room temperature (RT). Stained cells were stored in PBS at 4°C.

The immunostaining of cultured retinal explants was performed as described earlier.[56] The following primary antibodies were used: Anti-GFAP (1:1,000 dilution, catalog no. ab7260, Abcam, RRID: AB_305808), anti-PKCα (1:500, sc-8393, Santa Cruz, RRID: AB_628142), anti-mCherry (1: 500, ab205402, Abcam, RRID: AB_2722769), anti-Calbindin D28k (1:100, sc-365360, Santa Cruz, RRID: AB_10841576), anti-Laminin (LAMA1) (1:800, L9392, Sigma-Aldrich, RRID: AB_477163), and counterstained with 4′,6-diamidino-2-phenylindole (DAPI). The secondary antibodies used were: donkey anti-mouse 568 IgG (H + L, A10037, Thermo Fisher Scientific), goat anti-rabbit 488 IgG (H + L; A11008, Thermo Fisher Scientific), goat anti-mouse 647 IgG1 (γ1; A21240, Thermo Fisher Scientific), and goat anti-chicken 568 IgY (H + L; ab175477, Abcam), all used at a concentration of 1:1,000.

Image acquisition and analysis

Confocal micrographs were acquired using an LSM 710 confocal microscope (Zeiss, Oberkochen, Germany) as previously described.[56] For explants, volume scans covering the entire depth of the inner retina were acquired using a 40× oil immersion objective. The laser settings were kept constant for all images recorded per experimental series. In experiments aimed at quantifying the number of mCherry-positive cells or fluorescence intensity in cultured explants, adjacent volume scans were acquired across the entire explant—from one edge to the other—to capture a representative portion of the explant in its full depth. Care was taken to minimize any overlap between adjacent scans. For imaging of dissociated cells, a 20× water objective was used.

Image analysis was performed using ImageJ/Fiji (National Institute of Health, Bethesda, MD).[57] When stated, Neurite tracing of mCherry$^+$ cells was performed semi-automatically using the Simple Neurite Tracer (SNT) plugin.[58] Overlapping tiles were stitched in three dimensions with the pairwise stitching plugin[59] and the stitched image was z-projected with maximum intensity. For quantification of DAPI-positive cells, TrackMate was used to segment 3D images using the StarDist algorithm, and cell counts were obtained through automated analysis.[60] Post-processing was limited to global brightness and contrast adjustments, downscaling, cropping and selection of relevant fluorescent channels when appropriate.

Statistical analysis

Statistical analysis was performed using GraphPad Prism version 8 (GraphPad Software, San Diego, CA, USA). Data are presented as

mean ± SEM. For comparison between two groups, either a Mann-Whitney U test or an unpaired *t* test was performed as appropriate. Unless stated otherwise, comparisons involving more than two groups were analyzed using one-way ANOVA; when the ANOVA was significant, pairwise differences were tested with Tukey's HSD. Where comparing to a single control condition, Dunnett's test was used instead of Tukey's HSD. Statistical significance was denoted as $^{*}p < 0.05$, $^{**}p < 0.01$, $^{***}p < 0.001$, $^{****}p < 0.0001$.

DATA AND CODE AVAILABILITY

The datasets generated during and/or analyzed during the current study are available from the corresponding author on reasonable request.

ACKNOWLEDGMENTS

We would like to thank Enda McSweeny for his assistance in establishing retinal dissociate cell culture protocol. This work is supported by Grants from the University Medical Centre Giessen und Marburg to M.L. (UKGM 15/2020) and Deutsche Förderprogramm für Augenheilkunde of Bayer Vital GmbH to S.B. and M.L. Graphical abstract was created in BioRender: Biswas, S. (2026) https://BioRender.com/2kjz6kt.

AUTHOR CONTRIBUTIONS

S.B., M.L., and V.R. participated in research design. S.B., P.S., E.C., and N.X. conducted the experiments and S.B. performed the data analysis. S.B., E.C., N.X., V.R., and M.L. wrote or contributed to the writing of the manuscript.

DECLARATION OF INTERESTS

This work has been supported by Bayer Vital GmbH. Bayer had no role in the design of the experiments. S.B. holds a European Patent on mRNA modification unrelated to this work (EP4085932A1).

SUPPLEMENTAL INFORMATION

Supplemental information can be found online at https://doi.org/10.1016/j.omtn.2026.102996.

REFERENCES

1. Hinsch, V.G., Boye, S.L., and Boye, S.E. (2025). A Comprehensive Review of Clinically Applied Adeno-Associated Virus-Based Gene Therapies for Ocular Disease. Hum. Gene Ther. *36*, 1301–1320. https://doi.org/10.1089/hum.2024.252.
2. Lindner, M., Gilhooley, M.J., Hughes, S., and Hankins, M.W. (2022/11/01). Optogenetics for visual restoration: From proof of principle to translational challenges. Prog. Retin. Eye Res. *91*, 101089. https://doi.org/10.1016/j.preteyeres.2022.101089.
3. U.S. Food and Drug Administration (2025). Approved Cellular and Gene Therapy Products. https://www.fda.gov/vaccines-blood-biologics/cellular-gene-therapy-products/approved-cellular-and-gene-therapy-products.
4. Bucher, K., Rodríguez-Bocanegra, E., Dauletbekov, D., and Fischer, M.D. (2021). Immune responses to retinal gene therapy using adeno-associated viral vectors - Implications for treatment success and safety. Prog. Retin. Eye Res. *83*, 100915. https://doi.org/10.1016/j.preteyeres.2020.100915.
5. Wu, Z., Yang, H., and Colosi, P. (2010). Effect of genome size on AAV vector packaging. Mol. Ther. *18*, 80–86. https://doi.org/10.1038/mt.2009.255.
6. Biber, J., Gandor, C., Becirovic, E., and Michalakis, S. (2025). Retina-directed gene therapy: Achievements and remaining challenges. Pharmacol. Ther. *271*, 108862. https://doi.org/10.1016/j.pharmthera.2025.108862.
7. Rouf, N.Z., Biswas, S., Tarannum, N., Oishee, L.M., and Muna, M.M. (2022). Demystifying mRNA vaccines: an emerging platform at the forefront of cryptic diseases. RNA Biol. *19*, 386–410. https://doi.org/10.1080/15476286.2022.2055923.
8. Adams, D., Gonzalez-Duarte, A., O'Riordan, W.D., Yang, C.-C., Ueda, M., Kristen, A.V., Tournev, I., Schmidt, H.H., Coelho, T., Berk, J.L., et al. (2018-07-05). Patisiran, an RNAi Therapeutic, for Hereditary Transthyretin Amyloidosis. N. Engl. J. Med. Overseas. Ed. *379*, 11–21. https://doi.org/10.1056/NEJMoa1716153.
9. Tsuchida, C.A., Wasko, K.M., Hamilton, J.R., and Doudna, J.A. (2024). Targeted nonviral delivery of genome editors in vivo. Proc. Natl. Acad. Sci. USA *121*, e2307796121. https://doi.org/10.1073/pnas.2307796121.
10. Wang, T., Yu, T., Liu, Q., Sung, T.C., and Higuchi, A. (2024). Lipid nanoparticle technology-mediated therapeutic gene manipulation in the eyes. Mol. Ther. Nucleic Acids *35*, 102236. https://doi.org/10.1016/j.omtn.2024.102236.
11. Ryals, R.C., Patel, S., Acosta, C., McKinney, M., Pennesi, M.E., and Sahay, G. (2020). The effects of PEGylation on LNP based mRNA delivery to the eye. PLoS One *15*, e0241006. https://doi.org/10.1371/journal.pone.0241006.
12. Chambers, C.Z., Soo, G.L., Engel, A.L., Glass, I.A., Frassetto, A., Martini, P.G.V., Cherry, T.J., and Cherry, T.J. (2024). Lipid Nanoparticle-Mediated Delivery of mRNA Into the Mouse and Human Retina and Other Ocular Tissues. Transl. Vis. Sci. Technol. *13*, 7. https://doi.org/10.1167/tvst.13.7.7.
13. Patel, S., Ryals, R.C., Weller, K.K., Pennesi, M.E., and Sahay, G. (2019). Lipid nanoparticles for delivery of messenger RNA to the back of the eye. J. Control. Release *303*, 91–100. https://doi.org/10.1016/j.jconrel.2019.04.015.
14. Gautam, M., Jozic, A., Su, G.L.N., Herrera-Barrera, M., Curtis, A., Arrizabalaga, S., Tschetter, W., Ryals, R.C., and Sahay, G. (2023). Lipid nanoparticles with PEG-variant surface modifications mediate genome editing in the mouse retina. Nat. Commun. *14*, 6468. https://doi.org/10.1038/s41467-023-42189-3.
15. Devoldere, J., Peynshaert, K., Dewitte, H., Vanhove, C., De Groef, L., Moons, L., Özcan, S.Y., Dalkara, D., De Smedt, S.C., and Remaut, K. (2019). Non-viral delivery of chemically modified mRNA to the retina: Subretinal versus intravitreal administration. J. Control. Release *307*, 315–330. https://doi.org/10.1016/j.jconrel.2019.06.042.
16. Eygeris, Y., Gupta, M., Kim, J., Jozic, A., Gautam, M., Renner, J., Nelson, D., Bloom, E., Tuttle, A., Stoddard, J., et al. (2024). Thiophene-based lipids for mRNA delivery to pulmonary and retinal tissues. Proc. Natl. Acad. Sci. USA *121*, e2307813120. https://doi.org/10.1073/pnas.2307813120.
17. Herrera-Barrera, M., Ryals, R.C., Gautam, M., Jozic, A., Landry, M., Korzun, T., Gupta, M., Acosta, C., Stoddard, J., Reynaga, R., et al. (2023). Peptide-guided lipid nanoparticles deliver mRNA to the neural retina of rodents and nonhuman primates. Sci. Adv. *9*, eadd4623. https://doi.org/10.1126/sciadv.add4623.
18. Macosko, E.Z., Basu, A., Satija, R., Nemesh, J., Shekhar, K., Goldman, M., Tirosh, I., Bialas, A.R., Kamitaki, N., Martersteck, E.M., et al. (2015). Highly Parallel Genome-wide Expression Profiling of Individual Cells Using Nanoliter Droplets. Cell *161*, 1202–1214. https://doi.org/10.1016/j.cell.2015.05.002.
19. Sethi, C.S., Lewis, G.P., Fisher, S.K., Leitner, W.P., Mann, D.L., Luthert, P.J., and Charteris, D.G. (2005/01/01). Glial Remodeling and Neural Plasticity in Human Retinal Detachment with Proliferative Vitreoretinopathy. Investig. Ophthalmol. Vis. Sci. *46*, 329. https://doi.org/10.1167/iovs.03-0518.
20. Bringmann, A., Wiedemann, P., Bringmann, A., and Wiedemann, P. (2009). Involvement of Müller glial cells in epiretinal membrane formation. Graefe's Archive for Clinical and Experimental Ophthalmology *247*, 7–247. https://doi.org/10.1007/s00417-009-1082-x.
21. Li, W., Vanluchene, H., Raes, L., Peynshaert, K., Veys, L., Hernández, S.G., De Lombaerde, E., De Geest, B.G., Sanders, N.N., Moons, L., et al. (2025/09/10). Efficacy versus immunogenicity of LNP-mediated delivery of mRNA and self-amplifying RNA upon intravitreal injection in the mouse eye. J. Control. Release *385*, 114027. https://doi.org/10.1016/j.jconrel.2025.114027.
22. Bringmann, A., Pannicke, T., Grosche, J., Francke, M., Wiedemann, P., Skatchkov, S.N., Osborne, N.N., and Reichenbach, A. (2006). Muller cells in the healthy and diseased retina. Prog. Retin. Eye Res. *25*, 397–424. https://doi.org/10.1016/j.preteyeres.2006.05.003.
23. De Clerck, K., De Coster, E., Paoletti, L., De Smedt, S., Remaut, K., Peynshaert, K., De Clerck, K., De Coster, E., Paoletti, L., De Smedt, S., et al. (2025). Strategic lipid nanoparticle design dictates retinal delivery post inner limiting membrane disruption. Drug Deliv. Transl. Res. *16*, 1860–1876. https://doi.org/10.1007/s13346-025-01987-6.
24. RC, R., S, P., C, A., M, M., ME, P., and G, S. (2020). The effects of PEGylation on LNP based mRNA delivery to the eye - PubMed. PLoS One *15*, e0241006. https://doi.org/10.1371/journal.pone.0241006.

25. Pang, J.J., Lauramore, A., Deng, W.T., Li, Q., Doyle, T.J., Chiodo, V., Li, J., and Hauswirth, W.W. (2008). Comparative analysis of in vivo and in vitro AAV vector transduction in the neonatal mouse retina: effects of serotype and site of administration. Vision Res. *48*, 377–385. https://doi.org/10.1016/j.visres.2007.08.009.

26. Bull, N.D., Johnson, T.V., Welsapar, G., DeKorver, N.W., Tomarev, S.I., and Martin, K.R. (2011/05/01). Use of an Adult Rat Retinal Explant Model for Screening of Potential Retinal Ganglion Cell Neuroprotective Therapies. Investig. Ophthalmol. Vis. Sci. *52*, 3309. https://doi.org/10.1167/iovs.10-6873.

27. Schnichels, S., Kiebler, T., Hurst, J., Maliha, A.M., Löscher, M., Dick, H.B., Bartz-Schmidt, K.-U., Joachim, S.C., Sven Schnichels, T.K., Hurst, J., et al. (2019). Retinal Organ Cultures as Alternative Research Models. Altern. Lab. Anim. *47*, 19. https://doi.org/10.1177/0261192919840092.

28. Miguel, N.C.O., Matsuda, M., Portes, A.L.F., Allodi, S., Mendez-Otero, R., Puntar, T., Sholl-Franco, A., Krempel, P.G., and Monteiro, M.L.R. (2012/11/01). In Vitro Effects of Bevacizumab Treatment on Newborn Rat Retinal Cell Proliferation, Death, and Differentiation. Investig. Ophthalmol. Vis. Sci. *53*, 7904. https://doi.org/10.1167/iovs.12-10283.

29. Voke, E., Arral, M.L., Squire, H.J., Lin, T.-J., Zheng, L., Coreas, R., Lui, A., Iavarone, A.T., Pinals, R.L., Whitehead, K.A., et al. (2025). Protein corona formed on lipid nanoparticles compromises delivery efficiency of mRNA cargo. Nat. Commun. *16*, 1–16. https://doi.org/10.1038/s41467-025-63726-2.

30. Tavakoli, S., Kari, O.K., Turunen, T., Lajunen, T., Schmitt, M., Lehtinen, J., Tasaka, F., Parkkila, P., Ndika, J., Viitala, T., et al. (2020). Diffusion and Protein Corona Formation of Lipid-Based Nanoparticles in the Vitreous Humor: Profiling and Pharmacokinetic Considerations. Mol. Pharm. *18*, 699–713. https://doi.org/10.1021/acs.molpharmaceut.0c00411.

31. Peynshaert, K., Devoldere, J., De Smedt, S.C., and Remaut, K. (2018). In vitro and ex vivo models to study drug delivery barriers in the posterior segment of the eye. Adv. Drug Deliv. Rev. *126*, 44–57. https://doi.org/10.1016/j.addr.2017.09.007.

32. Omri, S., Omri, B., Savoldelli, M., Jonet, L., Thillaye-Goldenberg, B., Thuret, G., Gain, P., Jeanny, J.C., Crisanti, P., and Behar-Cohen, F. (2010). The outer limiting membrane (OLM) revisited: clinical implications. Clin. Ophthalmol. *4*, 183–195. https://doi.org/10.2147/opth.s5901.

33. Bejarano-Escobar, R., Sánchez-Calderón, H., Otero-Arenas, J., Martín-Partido, G., and Francisco-Morcillo, J. (2017). Muller glia and phagocytosis of cell debris in retinal tissue. J. Anat. *231*, 471–483. https://doi.org/10.1111/joa.12653.

34. Sakami, S., Imanishi, Y., and Palczewski, K. (2019). Muller glia phagocytose dead photoreceptor cells in a mouse model of retinal degenerative disease. FASEB J. *33*, 3680–3692. https://doi.org/10.1096/fj.201801662R.

35. Dalkara, D., Kolstad, K.D., Caporale, N., Visel, M., Klimczak, R.R., Schaffer, D.V., and Flannery, J.G. (2009). Inner limiting membrane barriers to AAV-mediated retinal transduction from the vitreous. Mol. Ther. *17*, 2096–2102. https://doi.org/10.1038/mt.2009.181.

36. De Clerck, K., Accou, G., Sauvage, F., Braeckmans, K., De Smedt, S.C., Remaut, K., and Peynshaert, K. (2022). Photodisruption of the Inner Limiting Membrane: Exploring ICG Loaded Nanoparticles as Photosensitizers. Pharmaceutics *14*, 1716. https://doi.org/10.3390/pharmaceutics14081716.

37. Hippert, C., Graca, A.B., Barber, A.C., West, E.L., Smith, A.J., Ali, R.R., and Pearson, R.A. (2015). Muller glia activation in response to inherited retinal degeneration is highly varied and disease-specific. PLoS One *10*, e0120415. https://doi.org/10.1371/journal.pone.0120415.

38. Park, Y.H., Snook, J.D., Zhuang, I., Shen, G., and Frankfort, B.J. (2020). Optimized culture of retinal ganglion cells and amacrine cells from adult mice. PLoS One *15*, e0242426. https://doi.org/10.1371/journal.pone.0242426.

39. Tuma, J., Chen, Y.J., Collins, M.G., Paul, A., Li, J., Han, H., Sharma, R., Murthy, N., and Lee, H.Y. (2023). Lipid Nanoparticles Deliver mRNA to the Brain after an Intracerebral Injection. Biochemistry *62*, 3533–3547. https://doi.org/10.1021/acs.biochem.3c00371.

40. Liu, G.W., Guzman, E.B., Menon, N., and Langer, R.S. (2023). Lipid Nanoparticles for Nucleic Acid Delivery to Endothelial Cells. Pharm. Res. *40*, 3–25. https://doi.org/10.1007/s11095-023-03471-7.

41. Han, E.L., Tang, S., Kim, D., Murray, A.M., Swingle, K.L., Hamilton, A.G., Mrksich, K., Padilla, M.S., Palanki, R., Li, J.J., and Mitchell, M.J. (2025). Peptide-Functionalized Lipid Nanoparticles for Targeted Systemic mRNA Delivery to the Brain. Nano Lett. *25*, 800–810. https://doi.org/10.1021/acs.nanolett.4c05186.

42. Haque, A.A., Weinmann, P., Biswas, S., Handgretinger, R., Mezger, M., Kormann, M.S.D., and Antony, J.S. (2020/04/01). RNA ImmunoGenic Assay: Simple method for detecting immunogenicity of in vitro transcribed mRNA. Adv. Cell Gene Ther. *3*, e3850. https://doi.org/10.1002/acg2.79.

43. Vaidyanathan, S., Azizian, K.T., Haque, A.K.M.A., Henderson, J.M., Hendel, A., Shore, S., Antony, J.S., Hogrefe, R.I., Kormann, M.S.D., Porteus, M.H., and McCaffrey, A.P. (2018). Uridine Depletion and Chemical Modification Increase Cas9 mRNA Activity and Reduce Immunogenicity without HPLC Purification. Mol. Ther. Nucleic Acids *12*, 530–542. https://doi.org/10.1016/j.omtn.2018.06.010.

44. Xiao, Y., Muhuri, M., Li, S., Qin, W., Xu, G., Luo, L., Li, J., Letizia, A.J., Wang, S.K., Chan, Y.K., et al. (2019). Circumventing cellular immunity by miR142-mediated regulation sufficiently supports rAAV-delivered OVA expression without activating humoral immunity. JCI Insight *4*, e99052. https://doi.org/10.1172/jci.insight.99052.

45. Varin, J., Bouzidi, N., Gauvain, G., Joffrois, C., Desrosiers, M., Robert, C., De Sousa Dias, M.M., Neuillé, M., Michiels, C., Nassisi, M., et al. (2021). Substantial restoration of night vision in adult mice with congenital stationary night blindness. Mol. Ther. Methods Clin. Dev. *22*, 15–25. https://doi.org/10.1016/j.omtm.2021.05.008.

46. Xue, K., and MacLaren, R.E. (2018). Ocular gene therapy for choroideremia: clinical trials and future perspectives. Expert Rev. Ophthalmol. *13*, 129–138. https://doi.org/10.1080/17469899.2018.1475232.

47. Verma, M., Ozer, I., Xie, W., Gallagher, R., Teixeira, A., and Choy, M. (2023). The landscape for lipid-nanoparticle-based genomic medicines. Nat. Rev. Drug Discov. *22*, 349–350. https://doi.org/10.1038/d41573-023-00002-2.

48. Yao, K., Qiu, S., Wang, Y.V., Park, S.J.H., Mohns, E.J., Mehta, B., Liu, X., Chang, B., Zenisek, D., Crair, M.C., et al. (2018). Restoration of vision after de novo genesis of rod photoreceptors in mammalian retinas. Nature *560*, 484–488. https://doi.org/10.1038/s41586-018-0425-3.

49. Jorstad, N.L., Wilken, M.S., Grimes, W.N., Wohl, S.G., VandenBosch, L.S., Yoshimatsu, T., Wong, R.O., Rieke, F., Reh, T.A., Jorstad, N.L., et al. (2017). Stimulation of functional neuronal regeneration from Müller glia in adult mice. Nature *548*, 103–107. https://doi.org/10.1038/nature23283.

50. Poulsen, K., Hanna, K., Nieves, J., Nguyen, N., Sharma, P., Grishanin, R., Corbau, R., and Kiss, S. (2025). Nonclinical study of ixo-vec gene therapy for nAMD supports efficacy for a human dose of 6E10 vg/eye and staggered dosing of fellow eyes. Mol. Ther. Methods Clin. Dev. *33*, 101430. https://doi.org/10.1016/j.omtm.2025.101430.

51. Henderson, J., O'Callaghan, J., and Campbell, M. (2024). Gene therapy for glaucoma: Targeting key mechanisms. Vision Res. *225*, 108502. https://doi.org/10.1016/j.visres.2024.108502.

52. Hoy, S.M. (2025). Revakinagene Taroretcel: First Approval. Mol. Diagn. Ther. *29*, 553–561. https://doi.org/10.1007/s40291-025-00787-5.

53. Choi, W., and Kohane, D.S. (2024). Hybrid Nanoparticle-Hydrogel Systems for Drug Delivery Depots and Other Biomedical Applications. ACS Nano *18*, 22780–22792. https://doi.org/10.1021/acsnano.4c06888.

54. Vogel, A.B., Lambert, L., Kinnear, E., Busse, D., Erbar, S., Reuter, K.C., Wicke, L., Perkovic, M., Beissert, T., Haas, H., et al. (2018). Self-Amplifying RNA Vaccines Give Equivalent Protection against Influenza to mRNA Vaccines but at Much Lower Doses. Mol. Ther. *26*, 446–455. https://doi.org/10.1016/j.ymthe.2017.11.017.

55. Gilhooley, M.J., Hickey, D.G., Lindner, M., Palumaa, T., Hughes, S., Peirson, S.N., MacLaren, R.E., and Hankins, M.W. (2021). ON-bipolar cell gene expression during retinal degeneration: Implications for optogenetic visual restoration. Exp. Eye Res. *207*, 108553. https://doi.org/10.1016/j.exer.2021.108553.

56. Kinder, L., and Lindner, M. (2025/02/03). Expression of Osteopontin in M2 and M4 Intrinsically Photosensitive Retinal Ganglion Cells in the Mouse Retina. Investig. Ophthalmol. Vis. Sci. *66*, 14. https://doi.org/10.1167/iovs.66.2.14.

57. Schindelin, J., Arganda-Carreras, I., Frise, E., Kaynig, V., Longair, M., Pietzsch, T., Preibisch, S., Rueden, C., Saalfeld, S., Schmid, B., et al. (2012). Fiji: an open-source

platform for biological-image analysis. Nat. Methods *9*, 676–682. https://doi.org/10.1038/nmeth.2019.

58. Arshadi, C., Günther, U., Eddison, M., Harrington, K.I.S., Ferreira, T.A., Arshadi, C., Günther, U., Eddison, M., Harrington, K.I.S., and Ferreira, T.A. (2021). SNT: a unifying toolbox for quantification of neuronal anatomy. Nat. Methods *18*, 374–377. https://doi.org/10.1038/s41592-021-01105-7.

59. Preibisch, S., Saalfeld, S., and Tomancak, P. (2009/06/01). Globally optimal stitching of tiled 3D microscopic image acquisitions. Bioinformatics *25*, 1463–1465. https://doi.org/10.1093/bioinformatics/btp184.

60. Ershov, D., Phan, M.-S., Pylvänäinen, J.W., Rigaud, S.U., Le Blanc, L., Charles-Orszag, A., Conway, J.R.W., Laine, R.F., Roy, N.H., Bonazzi, D., et al. (2022). TrackMate 7: integrating state-of-the-art segmentation algorithms into tracking pipelines. Nat. Methods *19*, 7–19. https://doi.org/10.1038/s41592-022-01507-1.

OMTN, Volume *37*

Supplemental information

Lipid nanoparticles enable mRNA delivery

to diverse cell types of the inner Retina

Sumit Biswas, Eleonora Carpentiero, Nermina Xhaferri, Paula Streckenbach, Vijay Renigunta, and Moritz Lindner

Supplemental Information

Table S1: Summary of reported metrics in this study

**Experimental Condition**	**Route of delivery**	**Metric**	**Degenerate explants**	**Healthy explants**	**Healthy explants (ILM-peeled)**
Late transfection	Subretinal	$mCherry^+$ cells/FOVs	28.25 [±2.60] (n=3 biological replicates, 8 FOVs)	Not assessed	Not assessed
Acute transfection	Subretinal	$mCherry^+$ cells/FOVs	34.889 [±4.01] (n=3 biological replicates, 9 FOVs)	Not assessed	Not assessed
Late transfection	Subretinal	$GFAP^+$ $mCherry^+$ cells/FOVs	22.375 [±2.43] (n=3 biological replicates, 8 FOVs)	Not assessed	Not assessed
Acute transfection	Subretinal	$GFAP^+$ $mCherry^+$ cells/FOVs	26.33 [±3.76] (n=3 biological replicates, 9 FOVs)	Not assessed	Not assessed
Late transfection	Subretinal	$GFAP^-$ $mCherry^+$ cells/FOVs	5.875 [±0.64] (n=3 biological replicates, 8 FOVs)	Not assessed	Not assessed
Acute transfection	Subretinal	$GFAP^-$ $mCherry^+$ cells/FOVs	8.555 [±0.88] (n=3 biological replicates, 9 FOVs)	Not assessed	Not assessed
Acute transfection	Intravitreal	$mCherry^+$ cells/FOVs	13.444 [±1.79] (n=3 biological replicates, 27 FOVs)	4.583 [±0.80], (n=3 biological replicates, 24 FOVs)	8.515 [±0.92] (n=3 biological replicates, 33 FOVs)
Acute transfection	Subretinal	$mCherry^+$ cells/FOVs	32.444 [±4.16] (n=3 biological replicates, 27 FOVs)	7.90 [±0.82] (n=3 biological replicates, 28 FOVs)	Not assessed
Acute transfection	Intravitreal	GFAP MFI (A.U.)	15703.4 [±1019.91] (n=3 biological replicates, 27 FOVs)	1646.92 [±238.56] (n=3 biological replicates, 24 FOVs)	2870.45 [±302.29] (n=3 biological replicates, 34 FOVs)
Acute transfection	Subretinal	GFAP MFI (A.U.)	14850.1 [±1593.71] (n=3 biological replicates, 27 FOVs)	2653.59 [±267.44] (n=3 biological replicates, 29 FOVs)	Not assessed
Acute transfection	Untransfected controls (NTC)	GFAP MFI (A.U.)	8756.56 [±487.24] (n=3 biological replicates, 25 FOVs)	1304.04 [±174.57] (n=3 biological replicates, 24 FOVs)	Not assessed

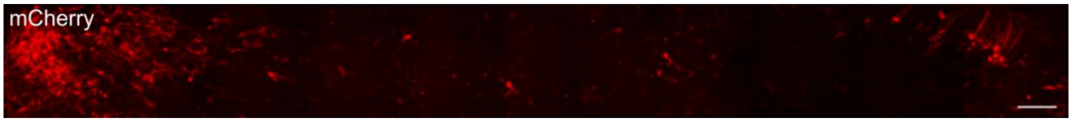

Figure S1: Comprehensive visualization of cmRNA^{mcherry}m^{1}Ψ$_{1.0}$m^{5}C$_{1.0}$-LNP transfected region in retina.

Retinal explants from rd1 retina-degenerate mice transfected by quasi-subretinal delivery (150 ng/µL). Multiple overlapping micrographs were stitched into a composite image to allow the assessment of transfection throughout the retinal explant. Scale bar: 150 µm.

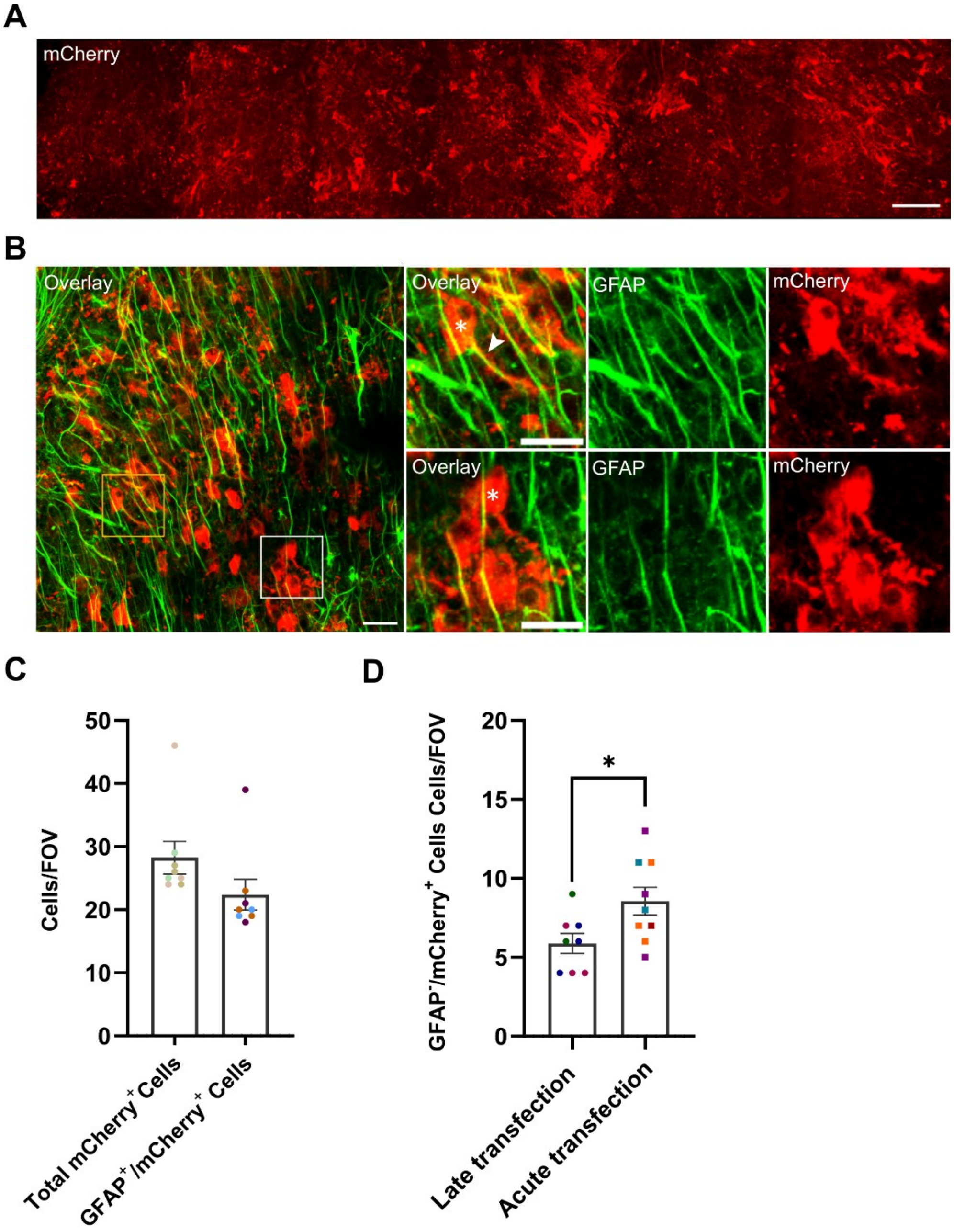

Figure S2: Late transfection in degenerate retinal explants using cmRNA^{mcherry}m$^{1}\Psi_{1.0}$m$^{5}C_{1.0}$-LNP

Degenerate retinal explants from rd1 mice transfected by quasi-subretinal delivery using LNPa two days after explantation. (A) Stitched composite image was generated from multiple overlapping micrographs to visualize the full extent of transfection across the explant. (B) Representative micrographs of explants immunostained for mCherry (red) and GFAP (green). Arrowhead represents the overlapping transfected cells and Muller glia process. Asterisk depicts a GFAP$^-$/mCherry$^+$ cell (C) Number of total mCherry$^+$ cells and subset of GFAP$^+$/mCherry$^+$ cells per field of view (FoV) after late transfection, assessed 24 hours post-transfection. (D) Quantification of GFAP$^-$/mCherry$^+$ cells per FoV, representing the complementary subset of the total mCherry$^+$ cells after late and acute

transfection. Dots represent data acquired from an individual FoV, colours represent individual biological replicates (3 per condition). A FoV represents a single confocal micrograph (212.55 µm x 212.55 µm). Note that GFAP does not stain the somata of Müller glia but rather their processes, hence, cells were graded as $GFAP^+$ based on their processes. Scale bar: A= 150 µm, B= 20 µm, B upper and lower panel= 10 µm. Data obtained from 3 independent biological replicates per condition. Statistical significance of the data in panel 2D was performed using an unpaired t test. Unless specified otherwise, data are shown as mean ± SEM. *p*-values are reported as ns (non-significant), * $p < 0.05$, ** $p < 0.01$, *** $p < 0.001$ or **** $p < 0.0001$.

A

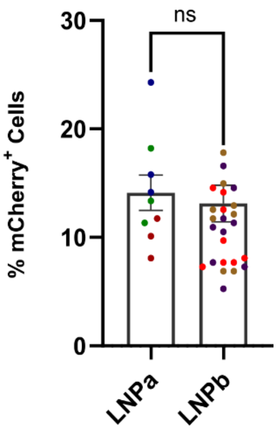

Figure S3: Transfection efficiency of LNPa and LNPb encapsulated cmRNA^{mcherry}m$^1\Psi_{1.0}$m^{5}C$_{1.0}$.

Degenerate retinas (rd1, C3H; A) were transfected with LNPa and LNPb using subretinal delivery under acute transfection conditions. Transfection efficiency was assessed 24 h post-transfection. Statistical significance was determined using Mann-Whitney U test. To enhance visualization, the y-axis in panel A is truncated; two values exceed the displayed range and are not shown but were included in the statistical analysis. Unless specified otherwise, data are shown as mean ± SEM. *p*-values are reported as ns (non-significant), * $p < 0.05$, ** $p < 0.01$, *** $p < 0.001$ or **** $p < 0.0001$.

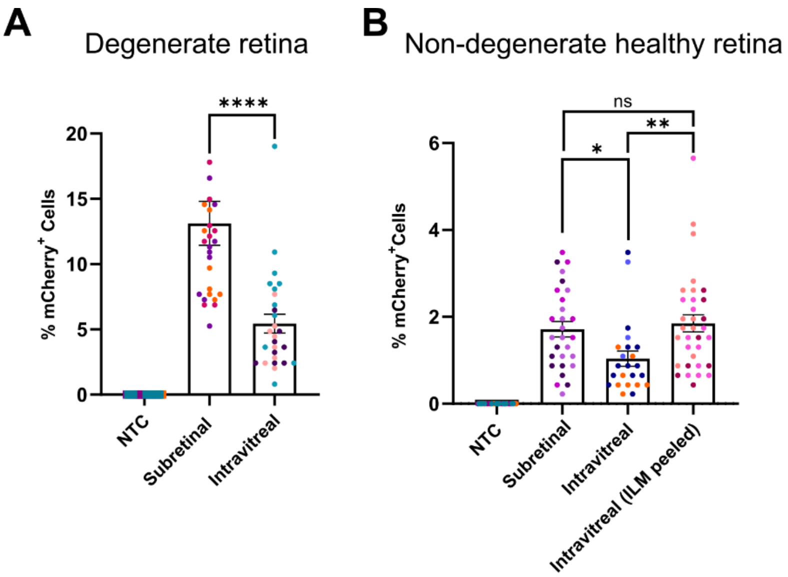

Figure S4: Transfection efficiency in degenerate and non-degenerate healthy retinal explants.

Quantification of transfection efficiency of cmRNA^{mcherry}m$^1\Psi_{1.0}$m^{5}C$_{1.0}$-LNP in retinal explants from degenerate (rd1, C3H; A) and non-degenerate healthy (C57BL/6J; B) mice following quasi-subretinal or intravitreal delivery of LNPs (150 ng/µL) was carried out 24 h post transfection. Non-treated control (NTC) retinas served as controls. Each dot represents a single field of view (FoV), and colors indicate individual biological replicates (n = 3 per condition). One FoV corresponds to a single confocal micrograph (212.55 µm × 212.55 µm). For visualization purposes, the y-axis in panel A is truncated; two values from the subretinal condition exceed the displayed range and are not shown but were included in the statistical analysis. The subretinal dataset in panel A corresponds to the LNPb condition shown in Figure S3. Data in panel A were analyzed using the Mann–Whitney U test, whereas data in panel B were analyzed using one-way ANOVA followed by Tukey's HSD post hoc test. Unless otherwise indicated, data are presented as mean ± SEM. Statistical significance is denoted as ns (not significant), *$p < 0.05$, **$p < 0.01$, ***$p < 0.001$, and ****$p < 0.0001$.

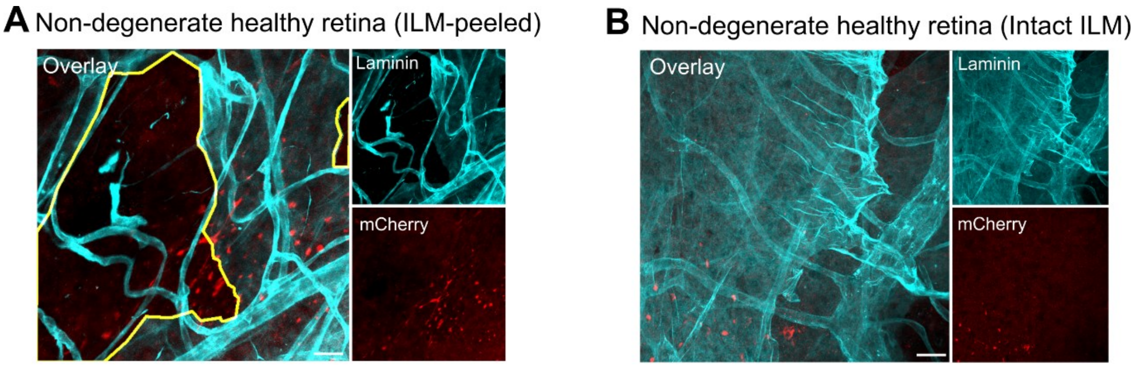

Figure S5: ILM-peeling promotes cellular uptake of cmRNA^{mcherry}m$^{1}\Psi_{1.0}$m^{5}C$_{1.0}$-LNP

ILM was partially removed during explantation of non-degenerate healthy explants (C57BL/6J). A representative confocal micrograph of (A) partially removed ILM and (B) intact ILM. Flat-mount retinal explants were double-stained with ILM marker laminin (cyan) and mCherry (red). ILM-peeled areas are highlighted (yellow). Scale bar: 20 µm.

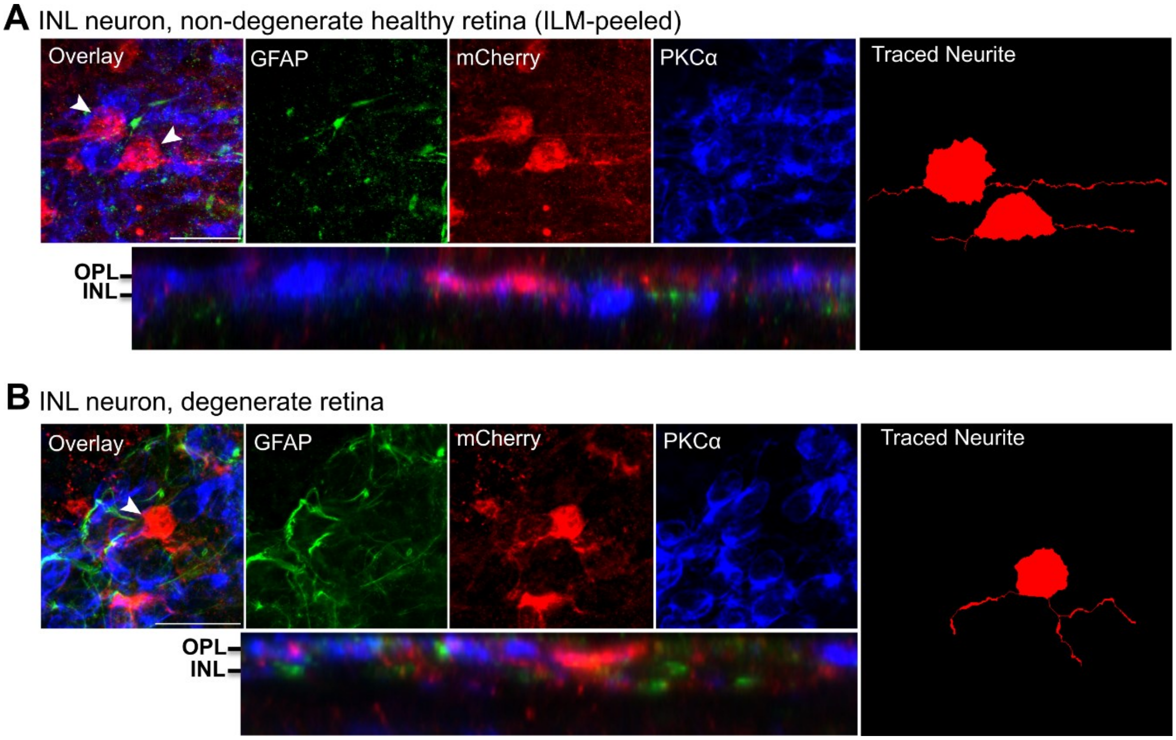

Figure S6: cmRNA^{mcherry}m$^1\Psi_{1.0}$m^{5}C$_{1.0}$-LNP transfects non-degenerate healthy and degenerate retinas.

Exemplary image from a high-resolution volume scan of (A) non-degenerate healthy explants (C57BL/6J) following intravitreal (ILM-peeled) and (B) degenerate explants (rd1, C3H) after subretinal administration. Flat-mount staining was performed using GFAP (green), mCherry (red) and PKCα (blue). Left panel: z-projection of the neuron-like structure with maximum intensity, with corresponding montage. Right panel: corresponding traced neurites. Arrowhead represents the associated traced neurite in the right panel. OPL: outer plexiform layer, INL: inner nuclear layer. Scale bar: 20 μm.

A

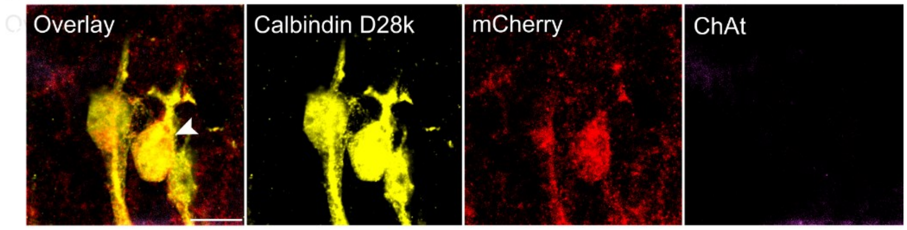

Figure S7: cmRNA^{mcherry}m^{1}Ψ$_{1.0}$m^{5}C$_{1.0}$-LNP transfects neuron.

Exemplary image from a high-resolution volume scan of (A) degenerate explants (rd1, C3H) following subretinal administration. Flat-mount staining was performed with Calbindin-D28K (yellow), mCherry (red) and ChAt (magenta). Arrow indicates overlap of mCherry and Calbindin-D28k within the soma. Scale bar: 10 µm.
